# Evaluating sampling strategy for DNA barcoding study of coastal and inland halo-tolerant Poaceae and Chenopodiaceae: A case study for increased sample size

**DOI:** 10.1371/journal.pone.0185311

**Published:** 2017-09-21

**Authors:** Peng-Cheng Yao, Hai-Yan Gao, Ya-Nan Wei, Jian-Hang Zhang, Xiao-Yong Chen, Hong-Qing Li

**Affiliations:** 1 School of Life Sciences, East China Normal University, Shanghai, China; 2 School of Ecological and Environmental Sciences, Tiantong National Station of Forest Ecosystem, East China Normal University, Shanghai, China; Istituto di Biologia e Biotecnologia Agraria Consiglio Nazionale delle Ricerche, ITALY

## Abstract

Environmental conditions in coastal salt marsh habitats have led to the development of specialist genetic adaptations. We evaluated six DNA barcode loci of the 53 species of Poaceae and 15 species of Chenopodiaceae from China's coastal salt marsh area and inland area. Our results indicate that the optimum DNA barcode was ITS for coastal salt-tolerant Poaceae and *matK* for the Chenopodiaceae. Sampling strategies for ten common species of Poaceae and Chenopodiaceae were analyzed according to optimum barcode. We found that by increasing the number of samples collected from the coastal salt marsh area on the basis of inland samples, the number of haplotypes of *Arundinella hirta*, *Digitaria ciliaris*, *Eleusine indica*, *Imperata cylindrica*, *Setaria viridis*, and *Chenopodium glaucum* increased, with a principal coordinate plot clearly showing increased distribution points. The results of a Mann-Whitney test showed that for *Digitaria ciliaris*, *Eleusine indica*, *Imperata cylindrica*, and *Setaria viridis*, the distribution of intraspecific genetic distances was significantly different when samples from the coastal salt marsh area were included (P < 0.01). These results suggest that increasing the sample size in specialist habitats can improve measurements of intraspecific genetic diversity, and will have a positive effect on the application of the DNA barcodes in widely distributed species. The results of random sampling showed that when sample size reached 11 for *Chloris virgata*, *Chenopodium glaucum*, and *Dysphania ambrosioides*, 13 for *Setaria viridis*, and 15 for *Eleusine indica*, *Imperata cylindrica* and *Chenopodium album*, average intraspecific distance tended to reach stability. These results indicate that the sample size for DNA barcode of globally distributed species should be increased to 11–15.

## Introduction

Since 2003, the concept of DNA barcoding has attracted the attention of botanical scientists from all over the world [1; 2; 3; 4; 5; 6; 7]. The Plant Working Group of the Consortium for the Barcode of Life recommended *rbc*L and *mat*K as the core barcodes for plants [8]. Later, *ITS* and *trn*H-*psb*A were also recommended as barcodes for plants [9; 10]. The use of these four loci as plant DNA barcodes has become widely accepted. Some studies have concentrated on evaluating identification capability for specific groups using these four barcode loci [11; 12; 13; 14; 15; 16; 17; 18], and others have focused on the discovery of new markers suitable for given taxa [6; 19; 20]. However, most studies leave out consideration of ecological environmental influences on plant genetic differentiation. Desert, lime rock, coastal salt marsh, polar circle, alpine, and other unique habitats significantly affect the morphology and heredity of their native plant species. Meyer and Paulay [21] have analyzed the effects of sampling scale on intraspecific genetic distance. By comparing intraspecific genetic distances in different cases when selecting 2, 5 and 10 individual samples, they found that the average coalescent depth increased as sample size increased (from 0.0049 to 0.0057 and then to 0.0070). They recommend a sample size of 5–10 individuals for DNA barcoding [21]. Other investigators have adopted this recommendation due to research costs [6; 12; 18; 19; 22]. However, the average coalescent depth reflects the maximum variation within the species, which has a large degree of randomness. Expanding a sampling range and considering individuals from a special habitat is bound to result in an increased number of samples, which conflict with the recommended strategy. As a result, in order to optimize the accuracy of DNA barcode evaluation, the appropriate number of samples remains to be explored.

Coastal halo-tolerant plants have specialized strategies [23], and their morphological identification can be challenging [24; 25]. This reflects in a quite number of widely distributed species. However, they are often neglected in DNA barcode researches. In fact, present DNA barcode databases such as the Marine Barcode of Life do not include data from coastal halo-tolerant plants. For such species, it is likely that when the samples size is increased over variable geographic territory, intraspecific genetic distance will expand along with sampling range [26, 27, 28]. Studies have also shown that plants have different morphological and molecular diversity in arid habitats [29; 30]. However, studies have not been conducted on whether or not the DNA barcode sampling of widely distributed plant species should specifically consider special ecological environments. Of the coastal halo-tolerant plants, Poaceae and Chenopodiaceae are the two largest families [31]. Poaceae are widely distributed globally [32], and are distributed in various ecological environments. Because of the difficulty in species identification, the importance of DNA barcode research in this family is self-evident. Some reports have included the DNA barcode of Poaceae [33; 34; 35; 36; 37], but none of these studies has specifically involved coastal halo-tolerant species. Chenopodiaceae include about 1700 species distributed in tropical and subtropical regions and well-adapted to arid environments [38]. Many species of this family live in inland saline and coastal salt marsh area [38]. While there are many studies on the phylogeny of Chenopodiaceae [39; 40; 41; 42; 43; 44], there are only 12 samples of 12 species of Chenopodiaceae reported by Bafeel for DNA barcode research [45]. The two families have a large number of widely distributed species that can grow coastally and inland, which provides an excellent model for a coastal/inland halo-tolerant plant DNA barcode comparative study.

## Materials and methods

### Samples

The silica gel samples together with vouchers were collected in non-protected areas for the access of which no permits were needed (no specific permissions were required for these locations/activities and the field studies did not involve endangered or protected species.

Samples from 68 species distributed in China’s coastal salt marsh area (223 Poaceae and 144 Chenopodiaceae) and 32 samples from inland China (19 Poaceae and 13 Chenopodiaceae) were collected for barcode sequencing. The sequence data of 799 further samples from the same species were downloaded from GenBank. Downloaded sequences met the following criteria: 1. species identification was accurate and reliable; 2. sample collection location was non-coastal salt marsh, or without collection site records but from a widely distributed species; and 3. sequence information is complete and reliable according to the information in the Genbank and sequences blast. Samples from the inland salt marshes and from GenBank that met the requirements were grouped as inland halo-tolerant plant samples. Wherever possible, each species included more than five samples from coastal halo-tolerant populations more than 50 kilometers apart, though several species had fewer than 5 samples. Sequences of *mat*K, *rbc*L, *ITS* and *trn*H-*psb*A were analyzed. For Poaceae, sequences of *rps*16 and *ndh*F that are widely sequenced in this family [46; 47; 48; 49; 50] were added as candidate loci, with *Pharus latifolius* L. and *Joinvillea plicata* (Hook. f.) Newell & B. C. Stone as outgroups. For Chenopodiaceae, sequences of *trn*L-F and *atp*B-*rbc*L were added as candidate loci [41; 42; 51], with *Gypsophila oldhamiana* Miq. and *Silene gallica* L. as outgroups. All specimens were stored in the herbarium of East China Normal University (HSNU), with GenBank accession numbers given in Supplementary S1 and S2 Tables.

### Analysis

#### DNA extraction, PCR amplification, and sequencing

DNA was extracted from 10 mg dry weight of each sample using CTAB [52]. PCR amplification was carried out using a TaKaRa TP600 (TaKaRa Bio, Inc., Otsu, Shiga, Japan). Primers and PCR amplification systems are given in S3 Table. PCR products were sequenced using Sanger by Huagene, Shanghai, China.

#### Sequence alignment and phylogenetic analysis

The sequences returned by the sequencing company were spliced and edited using Seqman (DNASTAR package, Madison, WI, USA) [53], followed by a comparison with the sequences downloaded from GenBank using the MUSCLE function in MEGA5.0 [54] to obtain a sequence matrix for “best close match” and phylogenetic analysis. A “best close match” operation was performed in TAXONDNA (identifying the query when the closest sequence is within a distance threshold) with a threshold of 3% calculated by the pairwise summary function [55]. Phylogenetic analysis was performed using Bayesian methods, model GTR+I+R for all the six loci of Poaceae and two loci (*ITS*, *trn*H-*psb*A) of Chenopodiaceae, GTR+G for *mat*K, *trn*L-F and *atp*B-*rbc*L of Chenopodiaceae, HKY+I for *rbc*L of Chenopodiaceae were selected under PAUP 4.0b10 and MrModelTest [56]. The tree was sampled every 1000 generations until the average deviation of split frequencies fell below 0.01 using MrBayes3.1.2 [57]. The species discrimination rate was calculated manually. When a branch achieved a supporting rate of over 95% in the Bayesian tree, it was defined as trustworthy. Comprehensive evaluation of the optimal barcodes was carried out for each of the two families.

#### Genetic diversity analysis

Haplotype analysis of the ten widespread species (1. *Arundinella hirta* (Thunb.) Tanaka, 2. *Chloris virgata* Sw., 3. *Dactyloctenium aegyptium* (L.) Beauv., 4. *Digitaria ciliaris* (Retz.) Koel., 5. *Eleusine indica* (L.) Gaertn., 6. *Imperata cylindrica* (L.) Beauv., 7. *Setaria viridis* (L.) Beauv., 8. *Chenopodium album* L., 9. *C*. *glaucum* L., 10. *Dysphania ambrosioides* (L.) Mosyakin & Clemants) was carried out by comparing the sequence matrices of the inland, coastal, and total samples using the MEGA 5.0 to obtain a K2P genetic distance matrix. A principal coordinate analysis was performed under GenALEx 6.5 [58]. Haplotype analysis was performed in DNAsp5.10.01 [59]. To obtain haplotype number, Autosome or Chloroplast model was selected according to the location of markers. M-W tests were performed in SPSS 20 [60] using K2P genetic distance matrices of inland samples and of whole samples. Boxplots for inland, coastal, and whole samples were plotted in SPSS 20.

#### Analysis of the relationship between sample size and the representativeness of DNA barcodes

Seven species (2. *Chloris virgata*, 5. *Eleusine indica*, 6. *Imperata cylindrica*, 7. *Setaria viridis*, 8. *Chenopodium album*, 9. *C*. *glaucum*, 10. *Dysphania ambrosioides*) with 17 samples or more of the ten widely distributed species were included in an analysis of the relationship between sample size and barcode representativeness. We hypothesized that the obtained samples of these species adequately reflected all variants of the associated species. Of these, the sample size of *Chenopodium album* was too large and was simplified based on the proportion of samples per haplotype, leaving 23 samples. Theta (θ) values (average K2P distances between different individuals in each species) of seven widely distributed species were calculated using APE package [61] using random sampling. Sample sizes from 2 to the number collected were tested for each species, each sample size was randomly sampled 20 times, and the average values of the obtained θ matrix were used to produce a scatter plot. A trend line was plotted by taking the maximum average value of θ over 20 samplings.

Genetic distance matrices were obtained for the seven widely distributed species. The confidence interval of genetic distance was calculated in SPSS 20 [60], with confidence level set at 99.99%. The confidence interval was obtained and the graph was merged with the scatter plot and trend line.

## Results

### Species differentiation rate of DNA barcodes for Chenopodiaceae and Poaceae

The Poaceae yielded 1233 novel sequences from 53 species (193 *ITS*, 215 *mat*K, 199 *rbc*L, 210 *trn*H-*psb*A, 226 *rps*16, 190 *ndh*F), and the Chenopodiaceae yielded 910 novel sequences from 15 species (150 *ITS*, 152 *mat*K, 147 *rbc*L, 152 *trn*H-*psb*A, 156 *trn*L-F, 153 *atp*B-*rbc*L). A total of 623 sequences from 53 species of Poaceae (337 *ITS*, 81 *rbc*L, 83 *mat*K, 53 *trn*H-*psb*A, 33 *rps*16, 36 *ndh*F) and 176 sequences from 15 species of Chenopodiaceae (66 *ITS*, 23 *rbc*L, 38 *mat*K, 27 *trn*H-*psb*A, 14 *trn*L-F, 8 *atp*B-*rbc*L) were selected from GenBank.

Sequence similarity analysis for Poaceae showed that the best discrimination occurs in *ITS* and *rps*16, with best close matches of 84.64% and 80.45%, respectively. Phylogenetic analysis showed that *ITS* (S1 Fig) and *mat*K showed a high discrimination with the resolution of 71.11% and 67.92% (Table 1). Sequence similarity results for Chenopodiaceae indicated that *mat*K and *trn*H-*psb*A showed the best results, with best close matches of 93.6% and 93.33%. Bayesian analysis indicated that the identification rates of *trn*L-F and *mat*K (S2 Fig) were relatively high, with the resolution of 86.67% and 80.00%, respectively (Table 1).

**Table 1:** Species discrimination on the basis of best close match and phylogenetic analysis.

Loci	Best close match (%)								Phylogenetic analysis(%)	
	Poaceae				Chenopodiaceae				Poaceae	Chenopodiaceae
	a	b	c	d	a	b	c	d		
*ITS*	84.64	11.23	2.62	1.49	82.53	14.81	0.52	2.11	71.11	73.33
*mat*K	77.25	20.73	2.0	0.0	93.6	5.81	0.58	0.0	67.92	80.00
*rbc*L	70.56	25.53	3.19	0.7	57.64	42.35	0.0	0.0	62.00	66.67
*trn*H-*psb*A	66.91	30.48	2.6	0.0	93.33	2.22	2.77	1.66	42.59	73.33
*ndh*F	73.47	19.56	6.08	0.86					63.04	
*rps*16	80.45	17.24	2.29	0.0					56.86	
*trn*L-F					87.05	12.35	0.58	0.0		86.67
*atp*B-*rbc*L					72.22	26.54	1.23	0.0		73.33

Note: Grey area indicates specific loci for Poaceae; pink indicates loci for Chenopodiaceae. a, Correct; b, Ambiguous; c, Incorrect; d, NO ID.

### Haplotypes obtained according to the optimal barcode of the Poaceae and Chenopodiaceae

Sequence comparison were performed on each of the 10 species. The haplotype was counted in DNAsp using the optimal barcode, *ITS* for Poaceae and *mat*K for Chenopodiaceae. As shown in Table 2, the number of haplotypes of species 1, 4, 5, 6, 7 and 9 increased when samples from coastal salt marshes were added.

**Table 2:** Haplotype number of 10 species sampled in inland habitat, coastal salt marshes, and the combined area.

Species	Inland		Coastal salt marshes		The combined area	
	a	b	a	b	a	b
1. *Arundinella hirta*	7	2	4	2	11	3
2. *Chloris virgata*	18	4	5	1	23	4
3. *Dactyloctenium aegyptium*	4	3	8	1	12	3
4. *Digitaria ciliaris*	5	3	4	3	9	5
5. *Eleusine indica*	14	4	9	4	23	5
6. *Imperata cylindrica*	13	7	11	6	24	9
7. *Setaria viridis*	11	3	8	5	19	7
8. *Chenopodium album*	25	3	34	1	58	3
9. *Chenopodium glaucum*	6	4	13	5	17	7
10. *Dysphania ambrosioides*	8	4	10	1	18	4

Note: a, Sample size; b, Haplotype (number).

### Effect of adding salt marsh samples on the genetic diversity of widely distributed species

**Fig 1:**
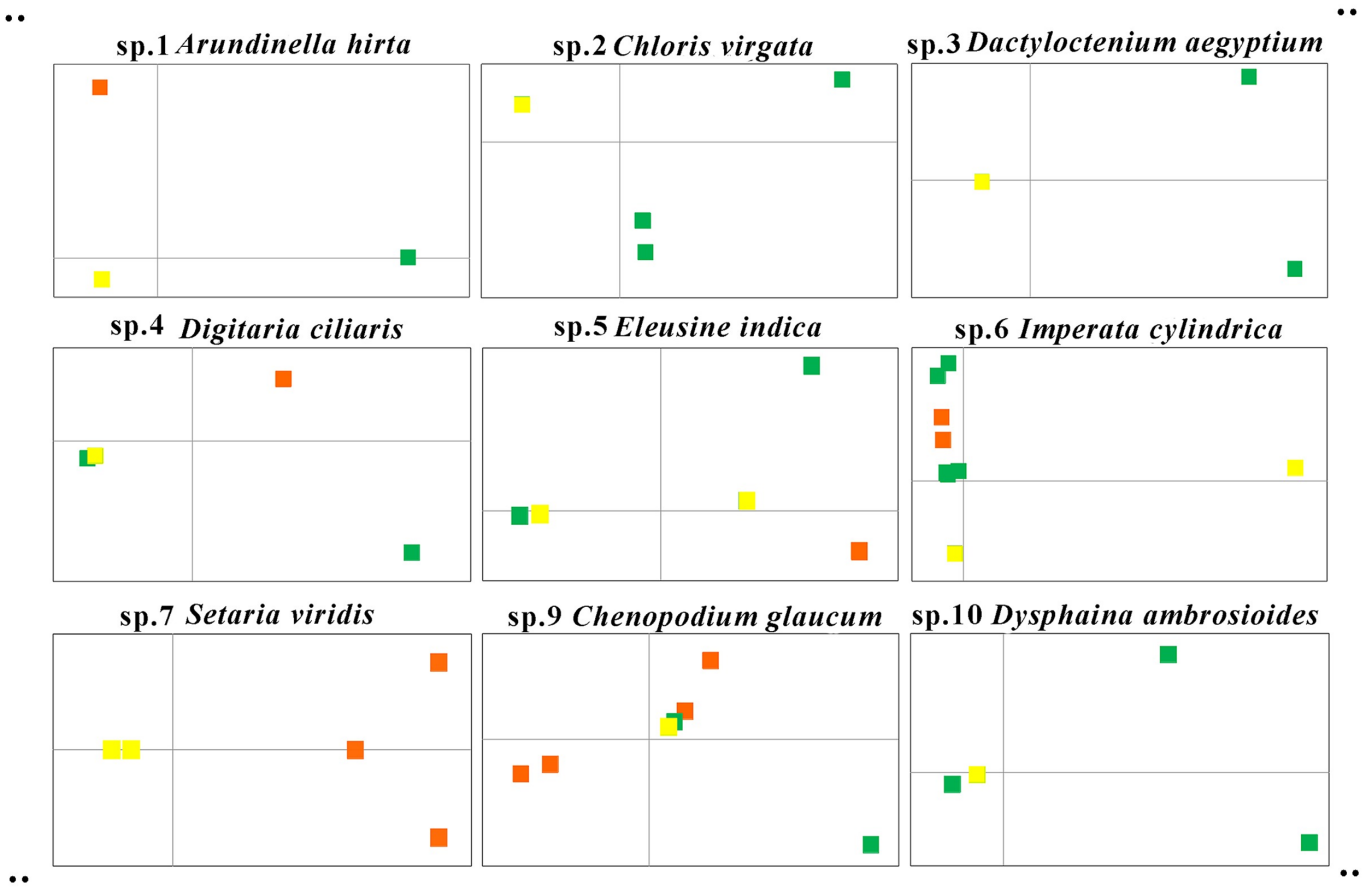
PCA Results of genetic distances variation when adding samples from coastal salt marshes. Green points represent samples from inland, orange points indicate samples from coastal salt marshes, and yellow points indicate samples from both inland and coastal salt marshes.

For species 8 (*Chenopodium album*), the principle component of the first dimension contributes a hundred percent due to the relatively small number of variable sites, so a two-dimensional PCA map cannot be made. The genetic diversity of the remaining nine widely distributed species was visualized using PCA (Fig 1). When the samples of coastal salt marsh were added, the species 1, 4, 5, 6, 7, and 9 showed obvious increased distribution points. Results were the same in the variation trend of the number of haplotypes.

### Intraspecific genetic distance distribution patterns in different sampling areas

**Fig 2:**
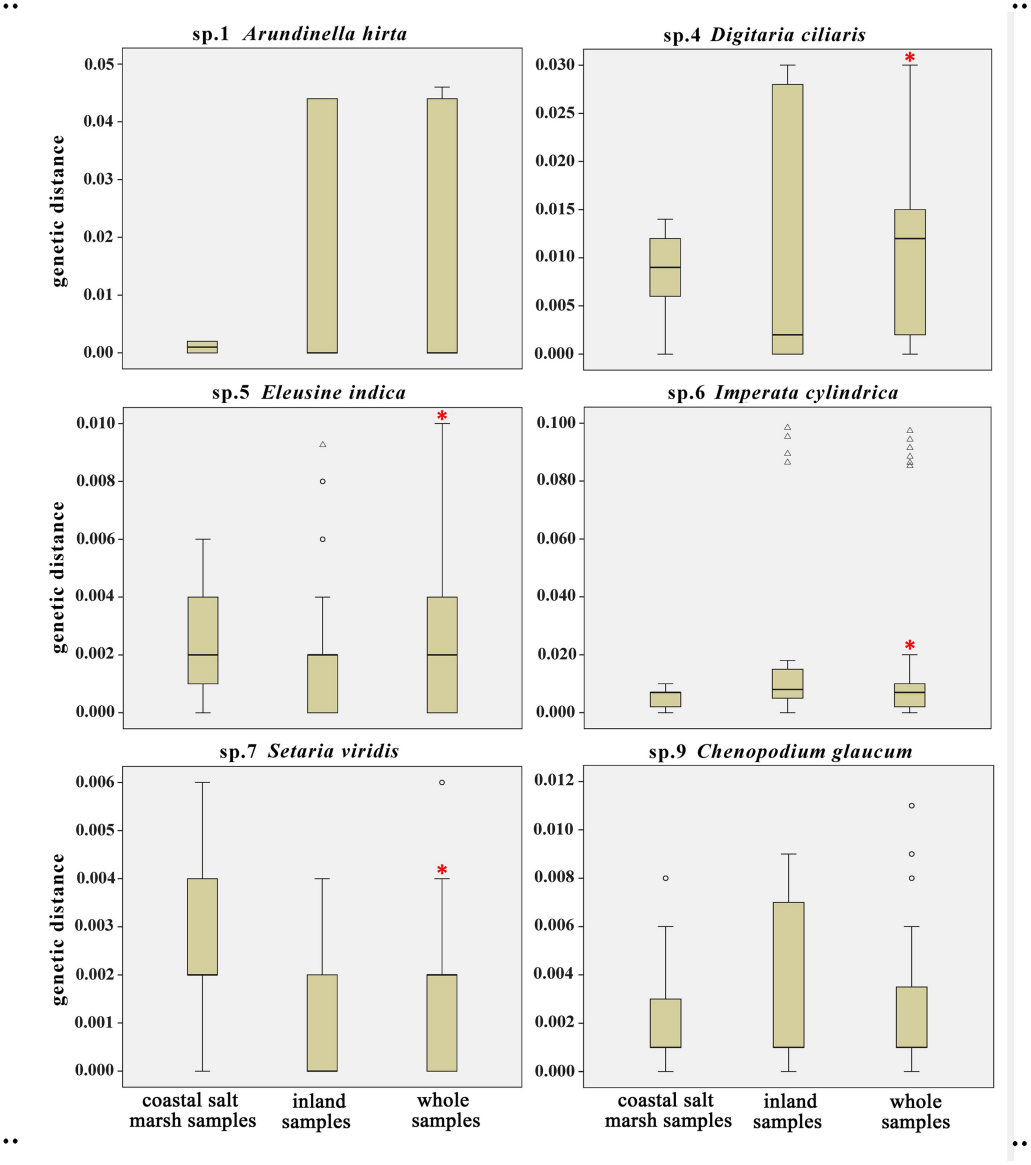
Genetic distance distribution of six widespread species and the results of M-W testing. Asterisk* indicates that samples from the combined area are significantly different from the inland samples in terms of genetic distance. Δ, ○ indicate outliers.

A Mann-Whitney test was performed and boxplots were constructed using the genetic distance matrices of the six widely distributed species, and showed an increase in the number of the haplotypes after adding the samples from the coastal salt marsh (Fig 2). These results indicate that the inclusion of coastal samples in the sample pool yielded significant differences in the intraspecific genetic distances of species 4–7 compared to inland samples only (P < 0.01). The boxplot of *Imperata* is more contracted because the variation of one sample was much bigger than that observed in all the others.

### Relationship between sample size and DNA barcoding data representativeness

**Fig 3:**
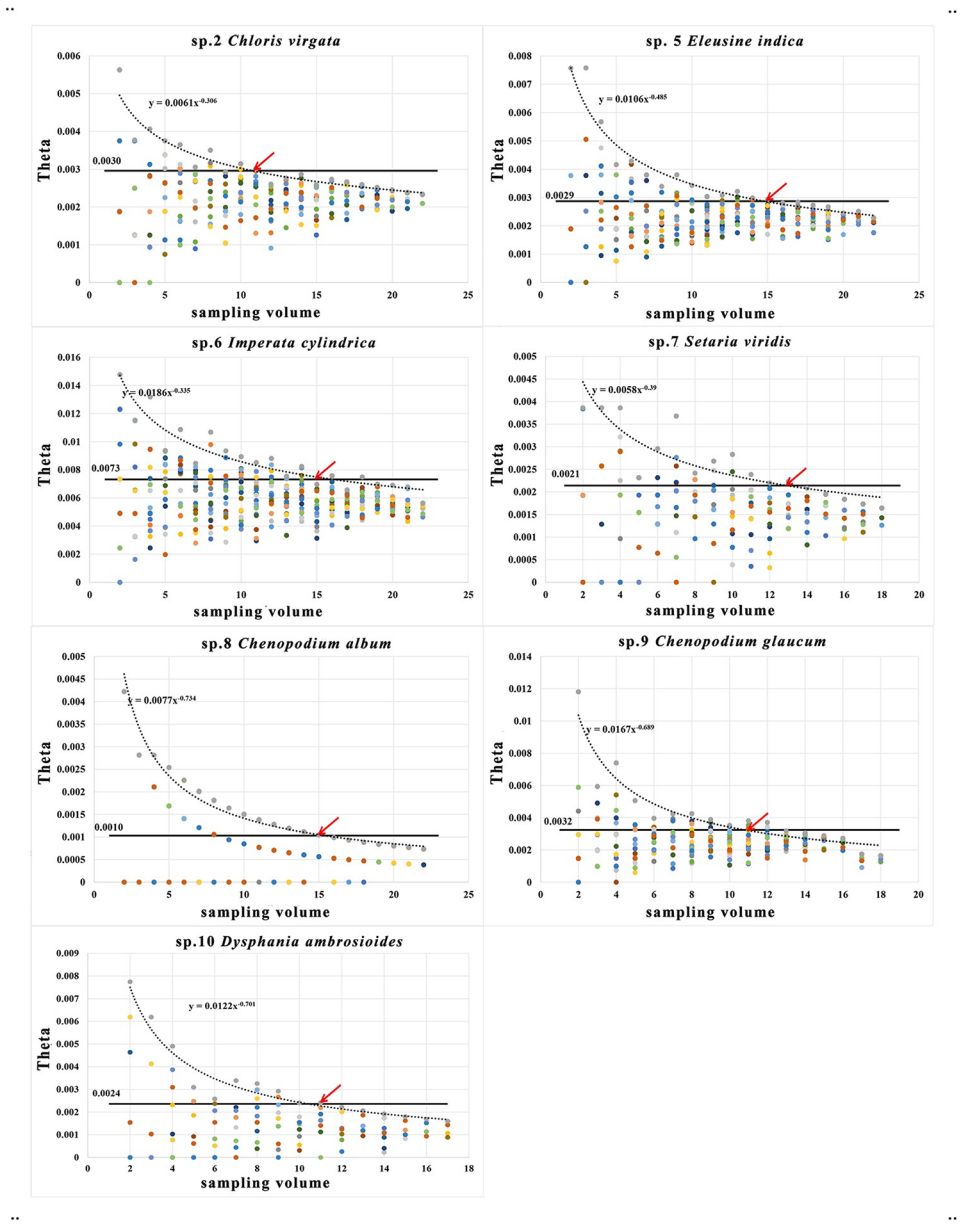
Theta (θ) of sampling volume for seven widespread species. The symbol—indicates the upper confidence interval at 99.99% confidence. The trend line is plotted by taking the maximum average value of θ at 20 replicates of each sampling. Red arrow indicates the minimum sampling volume when θ falls between the confidence intervals.

R language programming was used to calculate the effect of the sample size on the representativeness of DNA barcoding data (Fig 3). The distribution of θ for each species gradually converges to θ of all the samples as the sample size increases. When eleven samples were taken from species 2, 9 and 10, thirteen samples were taken from species 7, and fifteen samples were taken from species 5, 6 and 8, θ was less than the upper limit confidence interval of all samples. These results indicate that in the DNA barcode research for global distribution species, sample size should be expanded to 11–15.

## Discussion and conclusions

### *ITS* is the best DNA barcode for halo-tolerant Poaceae species in coastal areas

In the process of evaluating the DNA barcodes of the halo-tolerant Poaceae, both the best close match results based on sequence similarity and the phylogenetic analysis showed that discrimination using *ITS* was preferable to *mat*K (Table 1). Therefore, *ITS* is recommended as an optimal DNA barcode for halo-tolerant Poaceae species. This result is consistent with Peterson's findings in *Leptochloa* [62]. Although *ITS* was not at first the proposed optimal DNA barcode marker by the Consortium for the Barcode of Life, its evolution rate is three to four times that of plastid markers, and its application range has gradually exceeded that of *mat*K and *rbc*L [6; 16; 63]. Many taxonomic groups have been shown to be best represented by *ITS* as an optimum DNA barcode [11; 15; 18]. However, the limitations of study area and community in this investigation require that further research be conducted before *ITS* can be validated as applicable to Poaceae as a whole. The *trn*H-*psb*A sequences showed significant indels in the Poaceae, resulting in the lowest rate of discrimination. The candidate loci *rps*16 and *ndh*F have been widely used in phylogenetic studies of Poaceae [46; 47; 48; 49; 50]. However, we found that the discrimination rate of these two loci are considerably lower than that of *ITS*, and we discourage their use as DNA barcodes for the Poaceae.

### *Mat*K is the best DNA barcode for halo-tolerant Chenopodiaceae species in the coastal area

For the species of Chenopodiaceae in this study, there was no problem with amplification or primer universality for the six DNA barcode loci. In best close match analysis, *mat*K and *trn*H-*psb*A showed the best species discrimination rates. Bayesian tree analysis showed that *mat*K and *trn*L-F had similar discrimination rates (Table 1). The lengths of *trn*H-*psbA* sequences were relatively stable within the genera included in this study, but it is not clear whether they would remain stable when more genera are added. The resolution of *trn*L-F is positive in phylogenetic analysis [42; 64; 65], but is less than predicted by the best close match based on sequence similarity (Table 1), possibly due to its number of mutations leading to a within-species variation convergence rate below the threshold. Based on these evaluations, we suggest that *ma*tK is the optimal DNA barcode for coastal halo-tolerant Chenopodiaceae.

*Rbc*L has a high discrimination rate at the genus and family ranks, but has lower resolution within genus (Table 1), consistent with previous reports [4; 8; 10; 66; 67]. As an alternative, *ITS* and *mat*K could be used as substitutes when identifying genera and families [8; 68]. DNA barcodes of large genera, such as *Paphiopedilum* [12], *Ficus* [13], *Pedicularis* [18], and *Dendrobium* [69] have been evaluated, with findings supporting the used of *ITS* + *mat*K as a combined barcode for large genera. Since the object of DNA barcodes for identification is generally limited within genus, we suggest that the necessity of *rbc*L as a barcode for seed plants should be reevaluated.

### Saline habitat increases the genetic diversity of widespread species

Plants adapt with unique morphology and genetic differentiation in particular habitats [29, 30]. This study found significant genetic variation within Poaceae and Chenopodiaceae species distributed in coastal salt marsh areas compared with plants of the same species from other regions. This indicates an increase in genetic diversity of the species when coastal samples were added (Figs 1 and 2) and an increase in haplotypes within the species (Table 2). This is likely associated with coastal environmental conditions, including high salinity. These results indicate that when constructing the DNA barcode database of a species, samples from all kinds of habitats should be included [70]. While data on intraspecific and interspecific genetic distances obtained for locally distributed species [11; 12; 13; 14; 15; 16; 17; 18; 22] may be reliable, it is necessary to supplement sampling to make up for a lack of genetic diversity when considering widely distributed species.

### Sample size for DNA barcoding of widely distributed species should not be less than 11–15

The representativeness of DNA barcodes increases as sample size increases, and the expansion of the sampling range makes the evaluation of DNA barcodes more realistic [70]. Meyer & Paulay proposed strategies to take into account the cost of research, and suggested that sampling volume should limited to 5–10 individuals [21]. However, average K2P distances show that θ continuously converges as sample size increases, and θ falls into the confidence interval for all samples of a species when sample size is 11–15 (Fig 3). Our results indicate that the DNA barcode sampling of widespread species should not be less than 11–15, in order to accurately represent the extent of variation and genetic diversity. Using smaller sample sizes may lead to a significant loss of genetic diversity as shown in *Ficus simplicissima* Lour. (*s*.*l*.), where 5 additional haplotypes, based on the analysis of 78 samples, were added to the original 4 haplotypes base on 10 samples [13; 71]. By our experience, sampling of widely distributed species is relatively convenient, for the widely distributed species. The continuing decline in sequencing costs also helps make expanded sample sizes possible. Therefore, for widespread species, expanded sampling should not be cost-prohibitive and is to be encouraged when conducting barcode research. The difference in the minimum necessary sample size of different species may be related to the degree of intraspecific genetic differentiation, habitat diversity, distribution range.

## Supporting information

S1 Fig A tree of *ITS* sequences generated using MrBayes method of Poaceae. (PDF)

S2 Fig A tree of *mat*K sequences generated using MrBayes method of Chenopodiaceae. (PDF)

S1 Table Details of Poaceae material included in this study. (DOCX)

S2 Table Details of Chenopodiaceae material included in this study. (DOCX)

S3 Table Primers information and amplification protocol. (DOCX)
