# Supplementary figures and images for: Evaluating sampling strategy for DNA barcoding study of coastal and inland halo-tolerant Poaceae and Chenopodiaceae: A case study for increased sample size
Source: PLoS One. 2017 Sep 21;12(9):e0185311. doi: 10.1371/journal.pone.0185311 (PMC5608404; doi:10.1371/journal.pone.0185311)

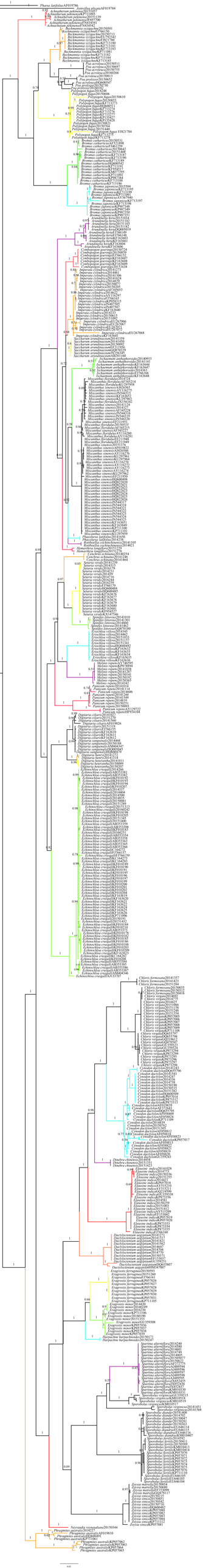

Supplement: S1 Fig — (PDF) [file pone.0185311.s001.pdf]

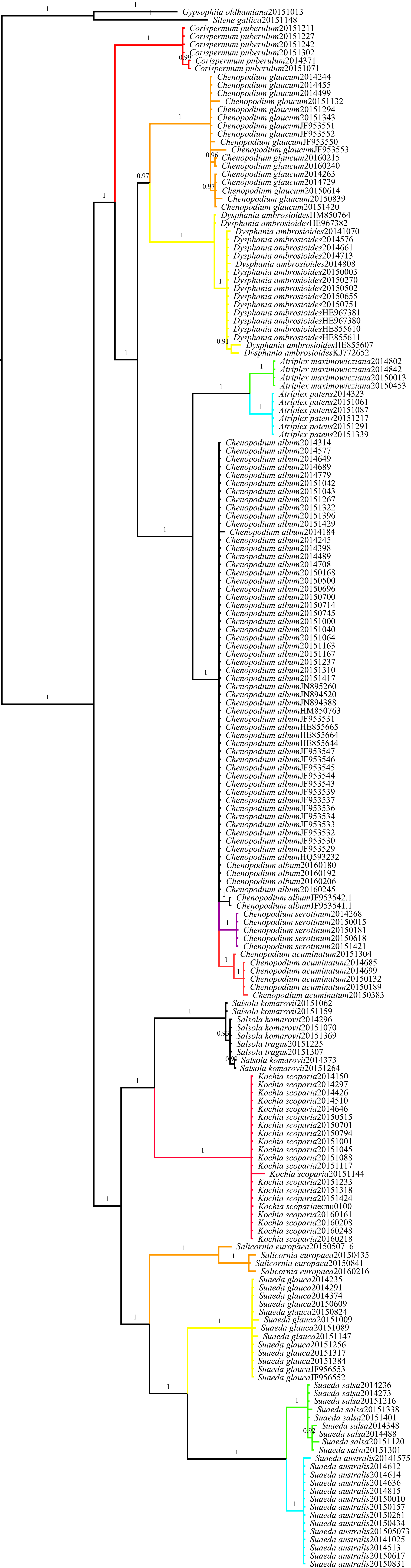

Supplement: S2 Fig — (PDF) [file pone.0185311.s002.pdf]
