# Supplementary material for: Evaluating sampling strategy for DNA barcoding study of coastal and inland halo-tolerant Poaceae and Chenopodiaceae: A case study for increased sample size
Source: PLoS One. 2017 Sep 21;12(9):e0185311. doi: 10.1371/journal.pone.0185311 (PMC5608404; doi:10.1371/journal.pone.0185311)
Supplement: S1 Table — (DOCX) [file pone.0185311.s003.docx]

**TableS1** Details of Poaceae material included in this study

| Taxon | Voucher Numbers | Collecter | Locality | GenBank accession | | | | | |
| --- | --- | --- | --- | --- | --- | --- | --- | --- | --- |
|  |  |  |  | *ITS* | *ma*tK | *rbc*L | *trn*H-*psb*A | *ndh*F | *rps*16 |
| *Achnatherum pekinense* (Hance) Ohwi | 20151057 | Yao,Li,Wei | Dalian Liaoning | MF063501 | MF064038 | - | MF064600 | MF065524 | MF073360 |
| *Achnatherum pekinense* (Hance) Ohwi | 20151139 | Yao,Li,Wei | Dalian Liaoning | MF063502 | MF064039 | - | MF064601 | MF065525 | MF073361 |
| *Achnatherum pekinense* (Hance) Ohwi |  |  |  | FN434541 | - | - | - | - | - |
| *Achnatherum pekinense* (Hance) Ohwi |  |  |  | FN434542 | - | - | - | - | - |
| *Achnatherum pekinense* (Hance) Ohwi |  |  |  | JF697700 | - | - | - | - | - |
| *Achnatherum pekinense* (Hance) Ohwi |  |  |  | KP711093 | - | - | - | - | - |
| *Arundinella hirta* (Thunb. ) Tanaka | 20151034 | Yao,Li,Wei | Dalian Liaoning | MF063503 | MF064040 | MF065147 | MF064602 | - | MF073362 |
| *Arundinella hirta* (Thunb. ) Tanaka | 20151102 | Yao,Li,Wei | Dalian Liaoning | MF063504 | MF064041 | MF065148 | MF064603 | - | - |
| *Arundinella hirta* (Thunb. ) Tanaka | 20151103 | Yao,Li,Wei | Dalian Liaoning | MF063505 | MF064042 | MF065149 | MF064604 | - | MF073363 |
| *Arundinella hirta* (Thunb. ) Tanaka | 20151182 | Yao,Li,Wei | Huludao Liaoning | MF063506 | MF064043 | MF065150 | MF064605 | - | MF073364 |
| *Arundinella hirta* (Thunb. ) Tanaka |  |  |  | DQ005019 | KF163709 | KC164297 | HQ600071 | - | LC117728 |
| *Arundinella hirta* (Thunb. ) Tanaka |  |  |  | FJ766148 | KF163710 | KF163493 | KP711183 | - | LC117743 |
| *Arundinella hirta* (Thunb. ) Tanaka |  |  |  | FJ766149 | KF163711 | KF163494 | - | - | LC117843 |
| *Arundinella hirta* (Thunb. ) Tanaka |  |  |  | KF163603 | KF163753 | KF163495 | - | - | LC117848 |
| *Arundinella hirta* (Thunb. ) Tanaka |  |  |  | KF163604 | - | KP711276 | - | - | LC117838 |
| *Arundinella hirta* (Thunb. ) Tanaka |  |  |  | KF163606 | - | - | - | - | - |
| *Arundinella hirta* (Thunb. ) Tanaka |  |  |  | KF163661 | - | - | - | - | - |
| *Arundo donax* L. | 2014172 | Li,Zhang | Sheshan Shanghai | - | - | - | MF064606 | - | MF073365 |
| *Arundo donax* L. | 20150537 | Yao, Wei | Wenzhou Zhejiang | - | MF064044 | MF065151 | MF064607 | - | MF073366 |
| *Arundo donax* L. | 20150703 | Yao, Wei | Wenling Zhejiang | - | MF064045 | MF065152 | - | - | - |
| *Arundo donax* L. | 20150840 | Yao, Wei | Zhoushan Zhejiang | - | MF064046 | - | MF064608 | - | MF073367 |
| *Beckmannia syzigachne* (Steud.) Fern. | 20150501 | Yao, Wei | Wenzhou Zhejiang | MF063507 | MF064047 | MF065153 | MF064609 | MF065526 | - |
| *Beckmannia syzigachne* (Steud.) Fern. | 20150713 | Yao, Wei | Yuhuan Zhejiang | MF063508 | MF064048 | MF065154 | MF064610 | MF065527 | - |
| *Beckmannia syzigachne* (Steud.) Fern. |  |  |  | EU792342 | KF713087 | EF125142 | KP711213 | DQ786817 | - |
| *Beckmannia syzigachne* (Steud.) Fern. |  |  |  | FJ766150 | KF713088 | KF712945 | - | HE575737 | - |
| *Beckmannia syzigachne* (Steud.) Fern. |  |  |  | FJ821780 | KF713089 | KF712946 | - | KM538735 | - |
| *Beckmannia syzigachne* (Steud.) Fern. |  |  |  | KF713180 | KF713090 | KF712947 | - | U71036.2 | - |
| *Beckmannia syzigachne* (Steud.) Fern. |  |  |  | KF713181 | KF713091 | KP711241 | - | - | - |
| *Beckmannia syzigachne* (Steud.) Fern. |  |  |  | KF713182 | - | - | - | - | - |
| *Beckmannia syzigachne* (Steud.) Fern. |  |  |  | KF713183 | - | - | - | - | - |
| *Beckmannia syzigachne* (Steud.) Fern. |  |  |  | KF713184 | - | - | - | - | - |
| *Beckmannia syzigachne* (Steud.) Fern. |  |  |  | KF713185 | - | - | - | - | - |
| *Beckmannia syzigachne* (Steud.) Fern. |  |  |  | KP711081 | - | - | - | - | - |
| *Bromus catharticus* Vahl | 20150531 | Yao, Wei | Wenzhou Zhejiang | MF063509 | MF064049 | MF065155 | MF064611 | MF065528 | MF073368 |
| *Bromus catharticus* Vahl | 20150642 | Yao, Wei | Wenling Zhejiang | MF063510 | MF064050 | MF065156 | MF064612 | MF065529 | MF073369 |
| *Bromus catharticus* Vahl | 20151408 | Yao, Wei, Yang | Yancheng Jiangsu | MF063511 | MF064051 | MF065157 | - | MF065530 | MF073370 |
| *Bromus catharticus* Vahl |  |  |  | AF521898 | KF713092 | KF712950 | HQ600129 | AY368048 | - |
| *Bromus catharticus* Vahl |  |  |  | FJ461259 | KF713093 | KF712951 | KF954532 | - | - |
| *Bromus catharticus* Vahl |  |  |  | HQ600542 | KF713094 | KF712952 | - | - | - |
| *Bromus catharticus* Vahl |  |  |  | KF713186 | KF713095 | KF712953 | - | - | - |
| *Bromus catharticus* Vahl |  |  |  | KF713187 | KJ772591 | KF712954 | - | - | - |
| *Bromus catharticus* Vahl |  |  |  | KF713188 | - | - | - | - | - |
| *Bromus catharticus* Vahl |  |  |  | KF713189 | - | - | - | - | - |
| *Bromus catharticus* Vahl |  |  |  | KF713190 | - | - | - | - | - |
| *Bromus catharticus* Vahl |  |  |  | KF713191 | - | - | - | - | - |
| *Bromus catharticus* Vahl |  |  |  | KF954517 | - | - | - | - | - |
| *Bromus catharticus* Vahl |  |  |  | KM077295 | - | - | - | - | - |
| *Bromus catharticus* Vahl |  |  |  | KP711092 | - | - | - | - | - |
| *Bromus catharticus* Vahl |  |  |  | KP987384 | - | - | - | - | - |
| *Bromus japonicus* Thunb. ex Murr. | 20150504 | Yao, Wei | Wenzhou Zhejiang | MF063512 | - | MF065158 | MF064613 | MF065531 | MF073371 |
| *Bromus japonicus* Thunb. ex Murr. |  |  |  | AY367940 | KF713098 | KF712960 | - | AY368034 | - |
| *Bromus japonicus* Thunb. ex Murr. |  |  |  | KF713195 | KF713099 | KF712961 | - | - | - |
| *Bromus japonicus* Thunb. ex Murr. |  |  |  | KF713197 | KF713100 | KF712962 | - | - | - |
| *Bromus japonicus* Thunb. ex Murr. |  |  |  | KF713198 | - | KF712963 | - | - | - |
| *Bromus japonicus* Thunb. ex Murr. |  |  |  | KF713199 | - | KP711251 | - | - | - |
| *Bromus japonicus* Thunb. ex Murr. |  |  |  | KP711091 | - | - | - | - | - |
| *Bromus japonicus* Thunb. ex Murr. |  |  |  | KP987348 | - | - | - | - | - |
| *Bromus japonicus* Thunb. ex Murr. |  |  |  | KP987349 | - | - | - | - | - |
| *Bromus japonicus* Thunb. ex Murr. |  |  |  | KP987350 | - | - | - | - | - |
| *Bromus japonicus* Thunb. ex Murr. |  |  |  | KP987351 | - | - | - | - | - |
| *Calamagrostis macrolepis* Litv. | 2014342 | Wei, Li, Zhang | Rongcheng Shandong | - | MF064052 | MF065159 | MF064614 | - | MF073372 |
| *Calamagrostis macrolepis* Litv. | 2014502 | Wei, Li, Zhang | Dongying Shandong | - | MF064053 | MF065160 | MF064615 | - | MF073373 |
| *Calamagrostis macrolepis* Litv. | 20151075 | Yao,Li,Wei | Dalian Liaoning | - | - | MF065161 | MF064616 | - | MF073374 |
| *Calamagrostis macrolepis* Litv. | 20151395 | Yao, Wei, Yang | Yancheng Jiangsu | - | - | MF065162 | MF064617 | - | MF073375 |
| *Cenchrus echinatus* L. | 20150254 | Yao, Wei | Huizhou Guangdong | MF063513 | MF064054 | MF065163 | MF064618 | MF065532 | MF073376 |
| *Cenchrus echinatus* L. | 20141246 | Yao, Li, Wei | Wenchang Hainan | MF063514 | MF064055 | MF065164 | - | MF065533 | MF073377 |
| *Cenchrus echinatus* L. | 20141329 | Yao, Li, Wei | Wanning Hainan | - | MF064056 | MF065165 | MF064619 | - | MF073378 |
| *Cenchrus echinatus* L. | 20141464 | Yao, Li, Wei | Lingshui Hainan | MF063515 | MF064057 | MF065166 | - | MF065534 | MF073379 |
| *Cenchrus echinatus* L. |  |  |  | - | KJ772641 | AM849359 | HG963728 | AF499151 | - |
| *Cenchrus echinatus* L. |  |  |  | - | - | - | - | HF558460 | - |
| *-Chloris formosana* (Honda) Keng | 20141357 | Yao, Li, Wei | Wanning Hainan | MF063516 | MF064058 | MF065167 | MF064620 | MF065535 | MF073380 |
| *Chloris formosana* (Honda) Keng | 20141423 | Yao, Li, Wei | Lingshui Hainan | MF063517 | MF064059 | MF065168 | MF064621 | MF065536 | MF073381 |
| *Chloris formosana* (Honda) Keng | 20150035 | Yao, Wei | Zhanjiang Guangdong | MF063518 | MF064060 | MF065169 | MF064622 | MF065537 | MF073382 |
| *Chloris formosana* (Honda) Keng | 20150312 | Yao, Wei | Huizhou Guangdong | MF063519 | MF064061 | MF065170 | MF064623 | MF065538 | MF073383 |
| *Chloris formosana* (Honda) Keng | 20150416 | Yao, Wei | Nan’ao Guangdong | MF063520 | MF064062 | MF065171 | MF064624 | MF065539 | MF073384 |
| *Chloris formosana* (Honda) Keng | 20151284 | Yao, Wei, Yang | Qinghuangdao Hebei | MF063521 | MF064063 | MF065172 | MF064625 | MF065540 | MF073385 |
| *Chloris virgata* Sw. | 2014425 | Wei, Li, Zhang | Penglai Shandong | MF063522 | MF064065 | MF065173 | MF064626 | MF065541 | MF073386 |
| *Chloris virgata* Sw. | 20151066 | Yao,Li,Wei | Dalian Liaoning | MF063523 | MF064066 | - | MF064627 | MF065542 | MF073387 |
| *Chloris virgata* Sw. | 20151158 | Yao,Li,Wei | Huludao Liaoning | MF063524 | MF064067 | MF065174 | MF064628 | MF065543 | MF073388 |
| *Chloris virgata* Sw. | 20151248 | Yao, Wei, Yang | Qinghuangdao Hebei | MF063525 | MF064068 | - | MF064629 | MF065544 | MF073389 |
| *Chloris virgata* Sw. | 20151354 | Yao, Wei, Yang | Lianyungang Jiangsu | MF063526 | MF064069 | MF065175 | MF064630 | MF065545 | MF073390 |
| *Chloris virgata* Sw. |  |  |  | DQ655797 | KP056918 | EF125096 | KP711188 | GU359726 | GQ219662 |
| *Chloris virgata* Sw. |  |  |  | DQ655798 | KP056919 | KP711264 | - | GU359727 | GU360443 |
| *Chloris virgata* Sw. |  |  |  | GQ219612 | KP056920 | - | - | - | GU360445 |
| *Chloris virgata* Sw. |  |  |  | GQ470547 | KP056921 | - | - | - | - |
| *Chloris virgata* Sw. |  |  |  | GU359323 | - | - | - | - | - |
| *Chloris virgata* Sw. |  |  |  | GU359324 | - | - | - | - | - |
| *Chloris virgata* Sw. |  |  |  | KP057005 | - | - | - | - | - |
| *Chloris virgata* Sw. |  |  |  | KP057006 | - | - | - | - | - |
| *Chloris virgata* Sw. |  |  |  | KP057007 | - | - | - | - | - |
| *Chloris virgata* Sw. |  |  |  | KP057008 | - | - | - | - | - |
| *Chloris virgata* Sw. |  |  |  | KP057009 | - | - | - | - | - |
| *Chloris virgata* Sw. |  |  |  | KP711108 | - | - | - | - | - |
| *Chloris virgata* Sw. |  |  |  | KP873294 | - | - | - | - | - |
| *Chloris virgata* Sw. |  |  |  | KP873295 | - | - | - | - | - |
| *Chloris virgata* Sw. |  |  |  | KP873296 | - | - | - | - | - |
| *Chloris virgata* Sw. |  |  |  | KP873297 | - | - | - | - | - |
| *Chloris virgata* Sw. |  |  |  | KP873298 | - | - | - | - | - |
| *Chloris virgata* Sw. |  |  |  | KP873299 | - | - | - | - | - |
| *Cymbopogon goeringii* (Steud.) A. Camus | 20141638 | Yao, Li, Wei | Changjiang Hainan | MF063527 | MF064070 | MF065176 | MF064631 | MF065546 | MF073391 |
| *Cymbopogon goeringii* (Steud.) A. Camus | 20150724 | Yao, Wei | Yuhuan Zhejiang | MF063528 | - | - | MF064632 | MF065547 | MF073392 |
| *Cymbopogon goeringii* (Steud.) A. Camus | 20150859 | Yao, Wei | Zhoushan Zhejiang | MF063529 | - | MF065177 | MF064633 | MF065548 | MF073393 |
| *Cymbopogon goeringii* (Steud.) A. Camus |  |  |  | FJ766152 | KF163712 | KC164298 | FJ766199 | - | - |
| *Cymbopogon goeringii* (Steud.) A. Camus |  |  |  | KF163607 | KF163713 | KF163496 | HQ600078 | - | - |
| *Cymbopogon goeringii* (Steud.) A. Camus |  |  |  | KF163608 | KF163714 | KF163497 | - | - | - |
| *Cymbopogon goeringii* (Steud.) A. Camus |  |  |  | KF163609 | - | - | - | - | - |
| *Cynodon dactylon* (L.) Pers. | 20141243 | Yao, Li, Wei | Wenchang Hainan | MF063530 | MF064071 | MF065178 | MF064634 | MF065549 | MF073394 |
| *Cynodon dactylon* (L.) Pers. | 20141543 | Yao, Li, Wei | Dongfang Hainan | MF063531 | MF064072 | MF065179 | MF064635 | MF065550 | - |
| *Cynodon dactylon* (L.) Pers. | 2014247 | Yao, Li, Wei | Cixi Zhejiang | MF063532 | MF064073 | MF065180 | MF064636 | MF065551 | MF073395 |
| *Cynodon dactylon* (L.) Pers. | 2014336 | Wei, Li, Zhang | Rongcheng Shandong | MF063533 | MF064074 | MF065181 | MF064637 | MF065552 | MF073396 |
| *Cynodon dactylon* (L.) Pers. | 2014738 | Wei, Li,Zhang* | Pingtan Fujian | MF063534 | MF064075 | MF065182 | MF064638 | MF065553 | MF073397 |
| *Cynodon dactylon* (L.) Pers. | 2014839 | Wei, Li,Zhang* | Zhangzhou Fujian | MF063535 | MF064076 | MF065183 | MF064639 | MF065554 | MF073398 |
| *Cynodon dactylon* (L.) Pers. | 20150190 | Yao, Wei | Huizhou Guangdong | MF063536 | MF064077 | - | MF064640 | MF065555 | MF073399 |
| *Cynodon dactylon* (L.) Pers. | 20150535 | Yao, Wei | Wenzhou Zhejiang | MF063537 | MF064078 | MF065184 | MF064641 | MF065556 | MF073400 |
| *Cynodon dactylon* (L.) Pers. | 20150762 | Yao, Wei | Zhoushan Zhejiang | MF063538 | MF064079 | MF065185 | MF064642 | MF065557 | MF073401 |
| *Cynodon dactylon* (L.) Pers. | 20151305 | Yao, Wei, Yang | Qinghuangdao Hebei | MF063539 | MF064080 | MF065186 | MF064643 | MF065558 | MF073402 |
| *Cynodon dactylon* (L.) Pers. | 20151382 | Yao, Wei, Yang | Lianyungang Jiangsu | MF063540 | MF064081 | MF065187 | MF064644 | MF065559 | MF073403 |
| *Cynodon dactylon* (L.) Pers. | 20160267 | Yao, Wei | Shihezi Xinjiang | - | MF064082 | MF065188 | - | MF065560 | MF073404 |
| *Cynodon dactylon* (L.) Pers. |  |  |  | AF019839 | AF144584 | AM849393 | EU531721 | AM849142 | - |
| *Cynodon dactylon* (L.) Pers. |  |  |  | AF058809 | JQ412229 | HM849930 | HE966594 | GU359603 | - |
| *Cynodon dactylon* (L.) Pers. |  |  |  | AF058812 | KJ772694 | KP056837 | HQ600101 | - | - |
| *Cynodon dactylon* (L.) Pers. |  |  |  | AF058815 | KP056930 | KP056840 | HQ876948 | - | - |
| *Cynodon dactylon* (L.) Pers. |  |  |  | AF058818 | KP149505 | KP711265 | KP711189 | - | - |
| *Cynodon dactylon* (L.) Pers. |  |  |  | AF058819 | - | - | - | - | - |
| *Cynodon dactylon* (L.) Pers. |  |  |  | AF058820 | - | - | - | - | - |
| *Cynodon dactylon* (L.) Pers. |  |  |  | AF058821 | - | - | - | - | - |
| *Cynodon dactylon* (L.) Pers. |  |  |  | AF058822 | - | - | - | - | - |
| *Cynodon dactylon* (L.) Pers. |  |  |  | AF058823 | - | - | - | - | - |
| *Cynodon dactylon* (L.) Pers. |  |  |  | AF058824 | - | - | - | - | - |
| *Cynodon dactylon* (L.) Pers. |  |  |  | AF372643 | - | - | - | - | - |
| *Cynodon dactylon* (L.) Pers. |  |  |  | DQ655795 | - | - | - | - | - |
| *Cynodon dactylon* (L.) Pers. |  |  |  | DQ655796 | - | - | - | - | - |
| *Cynodon dactylon* (L.) Pers. |  |  |  | GU359243 | - | - | - | - | - |
| *Cynodon dactylon* (L.) Pers. |  |  |  | HQ600509 | - | - | - | - | - |
| *Cynodon dactylon* (L.) Pers. |  |  |  | KP057014 | - | - | - | - | - |
| *Cynodon dactylon* (L.) Pers. |  |  |  | KP057017 | - | - | - | - | - |
| *Cynodon dactylon* (L.) Pers. |  |  |  | KP711109 | - | - | - | - | - |
| *Cynodon dactylon* (L.) Pers. |  |  |  | KP873312 | - | - | - | - | - |
| *Cynodon dactylon* (L.) Pers. |  |  |  | KP873313 | - | - | - | - | - |
| *Dactyloctenium aegyptium* (L.) Beauv. | 20141271 | Yao, Li, Wei | Wenchang Hainan | MF063541 | MF064083 | MF065189 | MF064645 | MF065561 | MF073405 |
| *Dactyloctenium aegyptium* (L.) Beauv. | 20141317 | Yao, Li, Wei | Wanning Hainan | MF063542 | MF064084 | MF065190 | MF064646 | MF065562 | MF073406 |
| *Dactyloctenium aegyptium* (L.) Beauv. | 20141421 | Yao, Li, Wei | Lingshui Hainan | MF063543 | MF064085 | MF065191 | MF064647 | MF065563 | MF073407 |
| *Dactyloctenium aegyptium* (L.) Beauv. | 20141562 | Yao, Li, Wei | Dongfang Hainan | MF063544 | MF064086 | MF065192 | MF064648 | MF065564 | MF073408 |
| *Dactyloctenium aegyptium* (L.) Beauv. | 2014647 | Wei, Li,Zhang* | Ningde Fujian | MF063545 | MF064087 | MF065193 | MF064649 | MF065565 | MF073409 |
| *Dactyloctenium aegyptium* (L.) Beauv. | 2014704 | Wei, Li,Zhang* | Pingtan Fujian | MF063546 | MF064088 | MF065194 | MF064650 | MF065566 | MF073410 |
| *Dactyloctenium aegyptium* (L.) Beauv. | 2014770 | Wei, Li,Zhang* | Zhangzhou Fujian | MF063547 | MF064089 | MF065195 | MF064651 | MF065567 | MF073411 |
| *Dactyloctenium aegyptium* (L.) Beauv. | 20150371 | Yao, Wei | Nan’ao Guangdong | MF063548 | - | - | - | MF065568 | MF073412 |
| *Dactyloctenium aegyptium* (L.) Beauv. |  |  |  | DQ655807 | JF729102 | EF125106 | HQ876949 | GU359713 | AY508682 |
| *Dactyloctenium aegyptium* (L.) Beauv. |  |  |  | EF153037 | KF357745 | - | - | HM346976 | GU360432 |
| *Dactyloctenium aegyptium* (L.) Beauv. |  |  |  | GU359251 | - | - | - | - | - |
| *Dactyloctenium aegyptium* (L.) Beauv. |  |  |  | HM347007 | - | - | - | - | - |
| *Digitaria ciliaris* (Retz.) Koel. | 20141027 | Yao, Zhang | Beihai Guangxi | MF063549 | MF064090 | MF065196 | MF064652 | MF065569 | MF073413 |
| *Digitaria ciliaris* (Retz.) Koel. | 20141326 | Yao, Li, Wei | Wanning Hainan | - | - | MF065197 | MF064653 | MF065570 | MF073414 |
| *Digitaria ciliaris* (Retz.) Koel. | 20141560 | Yao, Li, Wei | Dongfang Hainan | MF063550 | MF064091 | MF065198 | MF064654 | MF065571 | MF073415 |
| *Digitaria ciliaris* (Retz.) Koel. | 2014176 | Li,Zhang | Sheshan Shanghai | - | MF064092 | - | - | - | - |
| *Digitaria ciliaris* (Retz.) Koel. | 20150065 | Yao, Wei | Zhanjiang Guangdong | - | MF064093 | MF065199 | MF064655 | MF065572 | MF073416 |
| *Digitaria ciliaris* (Retz.) Koel. | 20151002 | Yao,Li,Wei | Dalian Liaoning | - | MF064094 | MF065200 | MF064656 | MF065573 | MF073417 |
| *Digitaria ciliaris* (Retz.) Koel. | 20151130 | Yao,Li,Wei | Dalian Liaoning | - | MF064095 | - | MF064657 | MF065574 | MF073418 |
| *Digitaria ciliaris* (Retz.) Koel. | 20151270 | Yao, Wei, Yang | Qinghuangdao Hebei | MF063551 | MF064096 | - | MF064658 | MF065575 | MF073419 |
| *Digitaria ciliaris* (Retz.) Koel. | 20151319 | Yao, Wei, Yang | Lianyungang Jiangsu | MF063552 | MF064097 | MF065201 | MF064659 | MF065576 | MF073420 |
| *Digitaria ciliaris* (Retz.) Koel. | 20151446 | Yao, Wei, Yang | Yancheng Jiangsu | - | MF064098 | MF065202 | MF064660 | MF065577 | MF073421 |
| *Digitaria ciliaris* (Retz.) Koel. |  |  |  | AF019826 | KF163808 | KC164299 | FJ766202 | AM849148 | LC118763 |
| *Digitaria ciliaris* (Retz.) Koel. |  |  |  | FJ766155 | KF163715 | KC164300 | KF263929 | - | LC118668 |
| *Digitaria ciliaris* (Retz.) Koel. |  |  |  | KF163610 | HE574067 | KC164301 | - | - | LC118748 |
| *Digitaria ciliaris* (Retz.) Koel. |  |  |  | KF163611 | LN906683 | KC164302 | - | - | LC118753 |
| *Digitaria ciliaris* (Retz.) Koel. |  |  |  | KF163612 | KF163823 | KF163498 | - | - | LC118758 |
| *Digitaria henryi* Rendle | 20141272 | Yao, Li, Wei | Wenchang Hainan | MF063553 | MF064099 | MF065203 | MF064661 | MF065578 | MF073422 |
| *Digitaria henryi* Rendle | 20141314 | Yao, Li, Wei | Wanning Hainan | MF063554 | MF064100 | - | MF064662 | MF065579 | MF073423 |
| *Digitaria heterantha* (Hook. f. ) Merr. | 20141011 | Yao, Zhang | Beihai Guangxi | MF063555 | MF064101 | MF065204 | MF064663 | MF065580 | MF073424 |
| *Digitaria heterantha* (Hook. f. ) Merr. | 20150089 | Yao, Wei | Zhanjiang Guangdong | MF063556 | MF064102 | MF065205 | MF064664 | MF065581 | MF073425 |
| *Digitaria heterantha* (Hook. f. ) Merr. | 20150207 | Yao, Wei | Huizhou Guangdong | MF063557 | MF064103 | MF065206 | MF064665 | MF065582 | MF073426 |
| *Digitaria sanguinalis* (L. ) Scop. | 2014468 | Wei, Li, Zhang | Penglai Shandong | MF063558 | MF064104 | MF065207 | MF064666 | MF065583 | MF073427 |
| *Digitaria sanguinalis* (L. ) Scop. | 20150188 | Yao, Wei | Huizhou Guangdong | MF063559 | MF064105 | MF065208 | MF064667 | - | MF073428 |
| *Digitaria sanguinalis* (L. ) Scop. |  |  |  | AM404347 | AF164421 | AJ746264 | HQ596673 | AM849147 | - |
| *Digitaria sanguinalis* (L. ) Scop. |  |  |  | DQ005045 | HM850571 | HM849953 | HQ600075 | - | - |
| *Digitaria sanguinalis* (L. ) Scop. |  |  |  | HQ600479 | HQ593271 | - | - | - | - |
| *Dinebra chinensis* (L.) Nees | 2014454 | Wei, Li, Zhang | Penglai Shandong | MF063560 | MF064106 | MF065209 | MF064668 | MF065584 | MF073429 |
| *Dinebra chinensis* (L.) Nees | 20151351 | Yao, Wei, Yang | Lianyungang Jiangsu | MF063561 | MF064107 | MF065210 | MF064669 | MF065585 | MF073430 |
| *Dinebra chinensis* (L.) Nees | 20151423 | Yao, Wei, Yang | Yancheng Jiangsu | MF063562 | - | MF065211 | MF064670 | MF065586 | MF073431 |
| *Dinebra chinensis* (L.) Nees |  |  |  | JQ345171 | - | - | - | - | - |
| *Dinebra chinensis* (L.) Nees |  |  |  | KP057057 | - | - | - | - | - |
| *Dinebra chinensis* (L.) Nees |  |  |  | KP057058 | - | - | - | - | - |
| *Echinochloa crusgalli* (L.) Beauv. | 2014266 | Wei, Zhang | Fengxian Shanghai | MF063563 | MF064108 | MF065212 | MF064671 | 2014266 | MF073432 |
| *Echinochloa crusgalli* (L.) Beauv. | 2014337 | Wei, Li, Zhang | Rongcheng Shandong | MF063564 | MF064109 | - | MF064672 | 2014337 | MF073433 |
| *Echinochloa crusgalli* (L.) Beauv. | 2014404 | Wei, Li, Zhang | Penglai Shandong | MF063565 | MF064110 | MF065213 | - | 2014404 | MF073434 |
| *Echinochloa crusgalli* (L.) Beauv. | 2014500 | Wei, Li, Zhang | Dongying Shandong | MF063566 | MF064111 | MF065214 | - | 2014500 | - |
| *Echinochloa crusgalli* (L.) Beauv. | 2014835 | Wei, Li,Zhang* | Zhangzhou Fujian | MF063567 | MF064112 | MF065215 | MF064673 | 2014835 | MF073435 |
| *Echinochloa crusgalli* (L.) Beauv. | 20150081 | Yao, Wei | Zhanjiang Guangdong | - | MF064113 | - | - | - | - |
| *Echinochloa crusgalli* (L.) Beauv. | 20150191 | Yao, Wei | Huizhou Guangdong | MF063568 | MF064114 | MF065216 | MF064674 | 20150191 | MF073436 |
| *Echinochloa crusgalli* (L.) Beauv. | 20151289 | Yao, Wei, Yang | Qinghuangdao Hebei | MF063569 | MF064115 | MF065217 | MF064675 | 20151289 | MF073437 |
| *Echinochloa crusgalli* (L.) Beauv. | 20151313 | Yao, Wei, Yang | Lianyungang Jiangsu | MF063570 | MF064116 | MF065218 | MF064676 | 20151313 | MF073438 |
| *Echinochloa crusgalli* (L.) Beauv. | 20151345 | Yao, Wei, Yang | Lianyungang Jiangsu | MF063571 | MF064117 | MF065219 | MF064677 | 20151345 | MF073439 |
| *Echinochloa crusgalli* (L.) Beauv. | 20151400 | Yao, Wei, Yang | Yancheng Jiangsu | MF063572 | MF064118 | MF065220 | MF064678 | 20151400 | MF073440 |
| *Echinochloa crusgalli* (L.) Beauv. | 20151432 | Yao, Wei, Yang | Yancheng Jiangsu | MF063573 | MF064119 | MF065221 | MF064679 | 20151432 | MF073441 |
| *Echinochloa crusgalli* (L.) Beauv. | 20160242 | Yao, Wei | Tulufan Xinjiang | - | - | - | - | - | MF073442 |
| *Echinochloa crusgalli* (L.) Beauv. | 20160251 | Yao, Wei | Shihezi Xinjiang | - | - | - | - | - | MF073443 |
| *Echinochloa crusgalli* (L.) Beauv. |  |  |  | AB353359 | - | KR058322 | GU575277 | AM849149 | - |
| *Echinochloa crusgalli* (L.) Beauv. |  |  |  | AB353381 | - | KR058324 | HQ596676 | - | - |
| *Echinochloa crusgalli* (L.) Beauv. |  |  |  | AB353398 | - | - | HQ600068 | - | - |
| *Echinochloa crusgalli* (L.) Beauv. |  |  |  | AB353354 | - | - | FJ766204 | - | - |
| *Echinochloa crusgalli* (L.) Beauv. |  |  |  | AB353358 | - | - | HQ600076 | - | - |
| *Echinochloa crusgalli* (L.) Beauv. |  |  |  | AB353363 | - | - | - | - | - |
| *Echinochloa crusgalli* (L.) Beauv. |  |  |  | AB353365 | - | - | - | - | - |
| *Echinochloa crusgalli* (L.) Beauv. |  |  |  | AB353366 | - | - | - | - | - |
| *Echinochloa crusgalli* (L.) Beauv. |  |  |  | AB353373 | - | - | - | - | - |
| *Echinochloa crusgalli* (L.) Beauv. |  |  |  | AB353382 | - | - | - | - | - |
| *Echinochloa crusgalli* (L.) Beauv. |  |  |  | AB353384 | - | - | - | - | - |
| *Echinochloa crusgalli* (L.) Beauv. |  |  |  | AB353385 | - | - | - | - | - |
| *Echinochloa crusgalli* (L.) Beauv. |  |  |  | AB353386 | - | - | - | - | - |
| *Echinochloa crusgalli* (L.) Beauv. |  |  |  | AB353387 | - | - | - | - | - |
| *Echinochloa crusgalli* (L.) Beauv. |  |  |  | AJ133707 | - | - | - | - | - |
| *Echinochloa crusgalli* (L.) Beauv. |  |  |  | AM404346 | - | - | - | - | - |
| *Echinochloa crusgalli* (L.) Beauv. |  |  |  | C164272 | - | - | - | - | - |
| *Echinochloa crusgalli* (L.) Beauv. |  |  |  | FJ766157 | - | - | - | - | - |
| *Echinochloa crusgalli* (L.) Beauv. |  |  |  | FJ766159 | - | - | - | - | - |
| *Echinochloa crusgalli* (L.) Beauv. |  |  |  | KC164273 | - | - | - | - | - |
| *Echinochloa crusgalli* (L.) Beauv. |  |  |  | KC164282 | - | - | - | - | - |
| *Echinochloa crusgalli* (L.) Beauv. |  |  |  | KC164283 | - | - | - | - | - |
| *Echinochloa crusgalli* (L.) Beauv. |  |  |  | KF010178 | - | - | - | - | - |
| *Echinochloa crusgalli* (L.) Beauv. |  |  |  | KF010182 | - | - | - | - | - |
| *Echinochloa crusgalli* (L.) Beauv. |  |  |  | KF010183 | - | - | - | - | - |
| *Echinochloa crusgalli* (L.) Beauv. |  |  |  | KF010184 | - | - | - | - | - |
| *Echinochloa crusgalli* (L.) Beauv. |  |  |  | KF010185 | - | - | - | - | - |
| *Echinochloa crusgalli* (L.) Beauv. |  |  |  | KF010186 | - | - | - | - | - |
| *Echinochloa crusgalli* (L.) Beauv. |  |  |  | KF010188 | - | - | - | - | - |
| *Echinochloa crusgalli* (L.) Beauv. |  |  |  | KF010189 | - | - | - | - | - |
| *Echinochloa crusgalli* (L.) Beauv. |  |  |  | KF010190 | - | - | - | - | - |
| *Echinochloa crusgalli* (L.) Beauv. |  |  |  | KF010191 | - | - | - | - | - |
| *Echinochloa crusgalli* (L.) Beauv. |  |  |  | KF010192 | - | - | - | - | - |
| *Echinochloa crusgalli* (L.) Beauv. |  |  |  | KF010193 | - | - | - | - | - |
| *Echinochloa crusgalli* (L.) Beauv. |  |  |  | KF010194 | - | - | - | - | - |
| *Echinochloa crusgalli* (L.) Beauv. |  |  |  | KF010195 | - | - | - | - | - |
| *Echinochloa crusgalli* (L.) Beauv. |  |  |  | KF010196 | - | - | - | - | - |
| *Echinochloa crusgalli* (L.) Beauv. |  |  |  | KF010197 | - | - | - | - | - |
| *Echinochloa crusgalli* (L.) Beauv. |  |  |  | KF010198 | - | - | - | - | - |
| *Echinochloa crusgalli* (L.) Beauv. |  |  |  | KF010199 | - | - | - | - | - |
| *Echinochloa crusgalli* (L.) Beauv. |  |  |  | KF010200 | - | - | - | - | - |
| *Echinochloa crusgalli* (L.) Beauv. |  |  |  | KF010201 | - | - | - | - | - |
| *Echinochloa crusgalli* (L.) Beauv. |  |  |  | KF010202 | - | - | - | - | - |
| *Echinochloa crusgalli* (L.) Beauv. |  |  |  | KF010203 | - | - | - | - | - |
| *Echinochloa crusgalli* (L.) Beauv. |  |  |  | KF010204 | - | - | - | - | - |
| *Echinochloa crusgalli* (L.) Beauv. |  |  |  | KF010205 | - | - | - | - | - |
| *Echinochloa crusgalli* (L.) Beauv. |  |  |  | KF010207 | - | - | - | - | - |
| *Echinochloa crusgalli* (L.) Beauv. |  |  |  | KF010208 | - | - | - | - | - |
| *Echinochloa crusgalli* (L.) Beauv. |  |  |  | KF010209 | - | - | - | - | - |
| *Echinochloa crusgalli* (L.) Beauv. |  |  |  | KF010210 | - | - | - | - | - |
| *Echinochloa crusgalli* (L.) Beauv. |  |  |  | KF163619 | - | - | - | - | - |
| *Echinochloa crusgalli* (L.) Beauv. |  |  |  | KF163620 | - | - | - | - | - |
| *Echinochloa crusgalli* (L.) Beauv. |  |  |  | KF163621 | - | - | - | - | - |
| *Echinochloa crusgalli* (L.) Beauv. |  |  |  | KF163622 | - | - | - | - | - |
| *Echinochloa crusgalli* (L.) Beauv. |  |  |  | KF163623 | - | - | - | - | - |
| *Echinochloa crusgalli* (L.) Beauv. |  |  |  | KF163624 | - | - | - | - | - |
| *Echinochloa crusgalli* (L.) Beauv. |  |  |  | KF163625 | - | - | - | - | - |
| *Echinochloa crusgalli* (L.) Beauv. |  |  |  | KF163626 | - | - | - | - | - |
| *Echinochloa crusgalli* (L.) Beauv. |  |  |  | KP711096 | - | - | - | - | - |
| *Eleusine indica* (L.) Gaertn. | 20141028 | Yao, Zhang | Beihai Guangxi | MF063574 | MF064120 | MF065222 | MF064680 | MF065598 | MF073444 |
| *Eleusine indica* (L.) Gaertn. | 2014421 | Wei, Li, Zhang | Penglai Shandong | MF063575 | MF064121 | - | MF064681 | MF065599 | MF073445 |
| *Eleusine indica* (L.) Gaertn. | 2014581 | Wei, Li,Zhang* | Ningde Fujian | MF063576 | MF064122 | MF065223 | MF064682 | - | MF073446 |
| *Eleusine indica* (L.) Gaertn. | 2014773 | Wei, Li,Zhang* | Zhangzhou Fujian | MF063577 | MF064123 | MF065224 | MF064683 | MF065600 | MF073447 |
| *Eleusine indica* (L.) Gaertn. | 20150259 | Yao, Wei | Huizhou Guangdong | MF063578 | MF064124 | MF065225 | MF064684 | MF065601 | MF073448 |
| *Eleusine indica* (L.) Gaertn. | 20150336 | Yao, Wei | Nan’ao Guangdong | MF063579 | MF064125 | MF065226 | MF064685 | MF065602 | MF073449 |
| *Eleusine indica* (L.) Gaertn. | 20151003 | Yao,Li,Wei | Dalian Liaoning | MF063580 | MF064126 | MF065227 | MF064686 | MF065603 | MF073450 |
| *Eleusine indica* (L.) Gaertn. | 20151255 | Yao, Wei, Yang | Qinghuangdao Hebei | MF063581 | MF064127 | MF065228 | MF064687 | MF065604 | MF073451 |
| *Eleusine indica* (L.) Gaertn. | 20151412 | Yao, Wei, Yang | Yancheng Jiangsu | MF063582 | MF064128 | MF065229 | MF064688 | MF065605 | MF073452 |
| *Eleusine indica* (L.) Gaertn. |  |  |  | KP057018 | KP056933 | AM887880 | FJ766207 | AM849151 | DQ242041 |
| *Eleusine indica* (L.) Gaertn. |  |  |  | AY515209 | KP056934 | HM849972 | GQ435009 | GU359698 | KP874040 |
| *Eleusine indica* (L.) Gaertn. |  |  |  | AY515210 | KP056935 | KP056842 | HQ600074 | KM538777 | KP874041 |
| *Eleusine indica* (L.) Gaertn. |  |  |  | AY515211 | KP056936 | KP056846 | - | - | LC115773 |
| *Eleusine indica* (L.) Gaertn. |  |  |  | EF153042 | - | KP056847 | - | - | - |
| *Eleusine indica* (L.) Gaertn. |  |  |  | FJ766160 | - | - | - | - | - |
| *Eleusine indica* (L.) Gaertn. |  |  |  | GQ219599 | - | - | - | - | - |
| *Eleusine indica* (L.) Gaertn. |  |  |  | GU359338 | - | - | - | - | - |
| *Eleusine indica* (L.) Gaertn. |  |  |  | KP057019 | - | - | - | - | - |
| *Eleusine indica* (L.) Gaertn. |  |  |  | KP057020 | - | - | - | - | - |
| *Eleusine indica* (L.) Gaertn. |  |  |  | KP873353 | - | - | - | - | - |
| *Eleusine indica* (L.) Gaertn. |  |  |  | KP873354 | - | - | - | - | - |
| *Eleusine indica* (L.) Gaertn. |  |  |  | KP873355 | - | - | - | - | - |
| *Eleusine indica* (L.) Gaertn. |  |  |  | KP873356 | - | - | - | - | - |
| *Elymus excelsus* Turcz. | 20151193 | Yao,Li,Wei | Huludao Liaoning | - | - | MF065230 | MF064689 | MF065606 | MF073453 |
| *Elymus excelsus* Turcz. | 20151300 | Yao, Wei, Yang | Qinghuangdao Hebei | - | MF064129 | MF065231 | MF064690 | MF065607 | MF073454 |
| *Elymus excelsus* Turcz. | 20160204 | Yao, Wei | Wulumuqi Xinjiang | - | MF064130 | MF065232 | - | - | MF073455 |
| *Elymus excelsus* Turcz. | 20160212 | Yao, Wei | Wulumuqi Xinjiang | - | MF064131 | MF065233 | MF064691 | MF065608 | MF073456 |
| *Elymus kamoji* (Ohwi) S. L. Chen | 2014254 | Yao, Li, Wei | Cixi Zhejiang | - | MF064132 | MF065234 | MF064692 | MF065609 | - |
| *Elymus kamoji* (Ohwi) S. L. Chen | 20150505 | Yao, Wei | Wenzhou Zhejiang | - | MF064133 | MF065235 | MF064693 | - | MF073457 |
| *Elymus kamoji* (Ohwi) S. L. Chen | 20150626 | Yao, Wei | Wenling Zhejiang | - | MF064134 | MF065236 | MF064694 | MF065610 | MF073458 |
| *Elymus kamoji* (Ohwi) S. L. Chen | 20160188 | Yao, Wei | Wulumuqi Xinjiang | - | - | MF065237 | MF064695 | - | MF073459 |
| *Elymus kamoji* (Ohwi) S. L. Chen | 20160201 | Yao, Wei | Wulumuqi Xinjiang | - | - | MF065238 | MF064696 | - | - |
| *Elymus kamoji* (Ohwi) S. L. Chen | 20160261 | Yao, Wei | Shihezi Xinjiang | - | - | MF065239 | MF064697 | - | MF073460 |
| *Eragrostis ferruginea* (Thunb.) Beauv. | 20150593 | Yao, Wei | Wenzhou Zhejiang | MF063583 | MF064135 | MF065241 | MF064698 | MF065611 | MF073461 |
| *Eragrostis ferruginea* (Thunb.) Beauv. | 20151063 | Yao,Li,Wei | Dalian Liaoning | - | MF064136 | MF065242 | MF064699 | MF065612 | MF073462 |
| *Eragrostis ferruginea* (Thunb.) Beauv. | 20151260 | Yao, Wei, Yang | Qinghuangdao Hebei | MF063584 | - | MF065243 | MF064700 | MF065613 | MF073463 |
| *Eragrostis ferruginea* (Thunb.) Beauv. |  |  |  | FJ766161 | KP056941 | KP056851 | HQ600070 | KM538778 | LC116018 |
| *Eragrostis ferruginea* (Thunb.) Beauv. |  |  |  | KP057026 | KP056942 | KP056853 | KP711173 | - | LC116023 |
| *Eragrostis ferruginea* (Thunb.) Beauv. |  |  |  | KP057027 | KP056943 | KP056854 | - | - | LC116028 |
| *Eragrostis ferruginea* (Thunb.) Beauv. |  |  |  | KP057028 | KP056944 | KP056855 | - | - | LC116033 |
| *Eragrostis ferruginea* (Thunb.) Beauv. |  |  |  | KP057029 | KP056945 | KP711258 | - | - | LC116038 |
| *Eragrostis ferruginea* (Thunb.) Beauv. |  |  |  | KP057030 | - | - | - | - | - |
| *Eragrostis ferruginea* (Thunb.) Beauv. |  |  |  | KP057031 | - | - | - | - | - |
| *Eragrostis ferruginea* (Thunb.) Beauv. |  |  |  | KP711105 | - | - | - | - | - |
| *Eragrostis minor* Host | 20151353 | Yao, Wei, Yang | Lianyungang Jiangsu | MF063586 | MF064137 | MF065246 | MF064705 | MF065615 | MF073468 |
| *Eragrostis minor* Host | 2014430 | Wei, Li, Zhang | Penglai Shandong | MF063585 | MF064138 | MF065245 | MF064704 | MF065614 | MF073467 |
| *Eragrostis minor* Host | 20160249 | Yao, Wei |  | MF063588 | MF064139 | MF065244 | MF064703 | MF065617 | MF073466 |
| *Eragrostis minor* (Thunb.) Beauv. | 20160190 | Yao, Wei | Wulumuqi Xinjiang | MF063587 | MF064140 | MF065240 | MF064701 | - | - |
| *Eragrostis minor* (Thunb.) Beauv. | 20160230 | Yao, Wei | Wulumuqi Xinjiang | MF063589 | MF064141 | MF065247 | MF064702 | MF065616 | MF073465 |
| *Eragrostis minor* Host |  |  |  | GU359308 | KM538801 | KP056861 | KM538899 | AM849154 | AY136851 |
| *Eragrostis minor* Host |  |  |  | KP057036 | KP056949 | KP056862 | - | GU359771 | GU360390 |
| *Eragrostis minor* Host |  |  |  | KP057037 | KP056950 | KP056863 | - | - | - |
| *Eragrostis minor* Host |  |  |  | KP057038 | KP056952 | KP711260 | - | - | - |
| *Eragrostis minor* Host |  |  |  | KP057039 | KP056953 | - | - | - | - |
| *Eragrostis minor* Host |  |  |  | KP711106 | - | - | - | - | - |
| *Eragrostis tenella* (L.) Beauv. ex Roem. et Schult. | 20141551 | Yao, Li, Wei | Dongfang Hainan | MF063634 | MF064142 | MF065248 | MF064706 | MF065618 | - |
| *Eragrostis tenella* (L.) Beauv. ex Roem. et Schult. | 20150098 | Yao, Wei | Zhanjiang Guangdong | - | MF064143 | MF065249 | MF064707 | MF065619 | MF073469 |
| *Eriochloa villosa* (Thunb.) Kunth | 2014345 | Wei, Li, Zhang | Rongcheng Shandong | MF063590 | MF064144 | MF065250 | MF064708 | MF065620 | MF073470 |
| *Eriochloa villosa* (Thunb.) Kunth | 2014462 | Wei, Li, Zhang | Penglai Shandong | MF063591 | MF064145 | MF065251 | MF064709 | MF065621 | MF073471 |
| *Eriochloa villosa* (Thunb.) Kunth | 20151033 | Yao,Li,Wei | Dalian Liaoning | MF063592 | MF064146 | MF065252 | MF064710 | MF065622 | MF073472 |
| *Eriochloa villosa* (Thunb.) Kunth | 20151157 | Yao,Li,Wei | Huludao Liaoning | MF063593 | MF064147 | MF065253 | MF064711 | MF065623 | MF073473 |
| *Eriochloa villosa* (Thunb.) Kunth | 20151341 | Yao, Wei, Yang | Lianyungang Jiangsu | MF063594 | MF064148 | MF065254 | MF064712 | MF065624 | MF073474 |
| *Eriochloa villosa* (Thunb.) Kunth |  |  |  | HQ600492 | KF163734 | KC164311 | HQ600089 | KM538704 | - |
| *Eriochloa villosa* (Thunb.) Kunth |  |  |  | KF163632 | KF163735 | KF163510 | - | - | - |
| *Eriochloa villosa* (Thunb.) Kunth |  |  |  | KF163633 | KF163736 | KF163511 | - | - | - |
| *Eriochloa villosa* (Thunb.) Kunth |  |  |  | KF163634 | KF163821 | KF163512 | - | - | - |
| *Eriochloa villosa* (Thunb.) Kunth |  |  |  | KF163635 | - | KF163513 | - | - | - |
| *Eriochloa villosa* (Thunb.) Kunth |  |  |  | KF163636 | - | - | - | - | - |
| *Harpachne harpachnoides* (Hack.) Keng | 20150171 | Yao, Wei | Zhanjiang Guangdong | MF063595 | - | - | MF064713 | - | MF073475 |
| *Harpachne harpachnoides* (Hack.) Keng | 20150247 | Yao, Wei | Huizhou Guangdong | MF063596 | MF064149 | MF065255 | MF064714 | MF065625 | MF073476 |
| *Harpachne harpachnoides* (Hack.) Keng |  |  |  | - | - | - | - | GU359734 | GU360382 |
| *Hemarthria longiflora* (Hook. f.) A. Camus | 20141317 | Yao, Li, Wei | Wanning Hainan | - | MF064150 | - | MF064715 | - | - |
| *Hemarthria longiflora* (Hook. f.) A. Camus | 20151229 | Yao,Li,Wei | Huludao Liaoning | MF063597 | - | MF065256 | MF064716 | - | MF073477 |
| *Hemarthria longiflora* (Hook. f.) A. Camus | 20151278 | Yao, Wei, Yang | Qinghuangdao Hebei | MF063598 | MF064151 | - | - | - | MF073478 |
| *Imperata cylindrica* (L.) Beauv. | 20141273 | Yao, Li, Wei | Wenchang Hainan | MF063599 | MF064152 | MF065257 | MF064717 | MF065626 | MF073479 |
| *Imperata cylindrica* (L.) Beauv. | 20141306 | Yao, Li, Wei | Wanning Hainan | MF063600 | MF064153 | MF065258 | MF064718 | MF065627 | MF073480 |
| *Imperata cylindrica* (L.) Beauv. | 20141624 | Yao, Li, Wei | Changjiang Hainan | MF063601 | - | - | MF064719 | MF065628 | MF073481 |
| *Imperata cylindrica* (L.) Beauv. | 2014233 | Yao, Li, Wei | Cixi Zhejiang | MF063602 | - | MF065259 | - | MF065629 | MF073482 |
| *Imperata cylindrica* (L.) Beauv. | 2014461 | Yao, Li, Wei | Wanning Hainan | MF063603 | MF064154 | MF065260 | - | MF065630 | MF073483 |
| *Imperata cylindrica* (L.) Beauv. | 2014622 | Wei, Li,Zhang* | Ningde Fujian | MF063604 | MF064155 | - | - | - | MF073484 |
| *Imperata cylindrica* (L.) Beauv. | 2014829 | Wei, Li,Zhang* | Zhangzhou Fujian | MF063605 | MF064156 | MF065261 | - | MF065631 | MF073485 |
| *Imperata cylindrica* (L.) Beauv. | 20150077 | Yao, Wei | Zhanjiang Guangdong | MF063606 | MF064157 | MF065262 | MF064720 | MF065632 | MF073486 |
| *Imperata cylindrica* (L.) Beauv. | 20150205 | Yao, Wei | Huizhou Guangdong | MF063607 | MF064158 | MF065263 | MF064721 | MF065633 | MF073487 |
| *Imperata cylindrica* (L.) Beauv. | 20150615 | Yao, Wei | Wenling Zhejiang | MF063608 | MF064159 | MF065264 | MF064722 | MF065634 | MF073488 |
| *Imperata cylindrica* (L.) Beauv. | 20151092 | Yao,Li,Wei | Dalian Liaoning | MF063609 | - | MF065265 | MF064723 | MF065635 | MF073489 |
| *Imperata cylindrica* (L.) Beauv. |  |  |  | AF345653 | GU135105 | AJ784826 | GU135443.2 | AM849161 | LC116653 |
| *Imperata cylindrica* (L.) Beauv. |  |  |  | AY116297 | KC123412 | KF163516 | JN407027.2 | - | LC116658 |
| *Imperata cylindrica* (L.) Beauv. |  |  |  | EU267066 | KF163737 | KF163517 | JN407028.2 | - | LC116663 |
| *Imperata cylindrica* (L.) Beauv. |  |  |  | EU267067 | KF163738 | - | JN407029.2 | - | LC116668 |
| *Imperata cylindrica* (L.) Beauv. |  |  |  | EU267068 | - | - | KP058330 | - | LC116673 |
| *Imperata cylindrica* (L.) Beauv. |  |  |  | EU267071 | - | - | - | - | - |
| *Imperata cylindrica* (L.) Beauv. |  |  |  | EU267072 | - | - | - | - | - |
| *Imperata cylindrica* (L.) Beauv. |  |  |  | FJ766165 | - | - | - | - | - |
| *Imperata cylindrica* (L.) Beauv. |  |  |  | JN407505 | - | - | - | - | - |
| *Imperata cylindrica* (L.) Beauv. |  |  |  | JN407507 | - | - | - | - | - |
| *Imperata cylindrica* (L.) Beauv. |  |  |  | KF163640 | - | - | - | - | - |
| *Imperata cylindrica* (L.) Beauv. |  |  |  | KF163641 | - | - | - | - | - |
| *Imperata cylindrica* (L.) Beauv. |  |  |  | KP058315 | - | - | - | - | - |
| *Ischaemum anthephoroides* (Steud.) Miq. | 2014091510 | Liu | Chongming Shanghai | MF063610 | MF064160 | MF065266 | MF064724 | MF065636 | MF073490 |
| *Ischaemum anthephoroides* (Steud.) Miq. | 20141141 | Yao, Zhang | Fangchenggang Guangxi | MF063611 | MF064161 | MF065267 | - | MF065637 | MF073491 |
| *Ischaemum anthephoroides* (Steud.) Miq. | 2014363 | Wei, Li, Zhang | Rongcheng Shandong | MF063612 | MF064162 | MF065268 | MF064725 | MF065638 | MF073492 |
| *Ischaemum anthephoroides* (Steud.) Miq. |  |  |  | FJ766166 | KF163810 | KC164315 | FJ766213 | KM538705 | - |
| *Ischaemum anthephoroides* (Steud.) Miq. |  |  |  | KF163644 | KF163815 | KC164316 | HQ600066 | - | - |
| *Ischaemum anthephoroides* (Steud.) Miq. |  |  |  | KF163647 | KF163822 | KF163519 | - | - | - |
| *Ischaemum anthephoroides* (Steud.) Miq. |  |  |  | KF163648 | - | - | - | - | - |
| *Ischaemum aristatum* L. | 20141628 | Yao, Li, Wei | Changjiang Hainan | - | MF064163 | MF065269 | MF064726 | MF065639 | MF073493 |
| *Leymus mollis* (Trin.) Hara | 2014300 | Wei, Li, Zhang | Rongcheng Shandong | - | MF064164 | MF065270 | MF064727 | MF065640 | MF073494 |
| *Leymus mollis* (Trin.) Hara | 20151199 | Yao,Li,Wei | Huludao Liaoning | - | MF064165 | MF065271 | MF064728 | MF065641 | MF073495 |
| *Melinis repens* (Willd.) Zizka | 20141038 | Yao, Zhang | Beihai Guangxi | MF063645 | MF064202 | MF065301 | MF064764 | - | MF073496 |
| *Melinis repens* (Willd.) Zizka | 20141287 | Yao, Li, Wei | Wenchang Hainan | MF063646 | MF064203 | MF065302 | MF064765 | MF065678 | MF073497 |
| *Melinis repens* (Willd.) Zizka | 2014342 | Wei, Li, Zhang | Rongcheng Shandong | MF063647 | MF064204 | MF065303 | MF064766 | MF065679 | MF073498 |
| *Melinis repens* (Willd.) Zizka | 20150141 | Yao, Wei | Zhanjiang Guangdong | MF063648 | MF064205 | MF065304 | MF064767 | MF065680 | MF073499 |
| *Melinis repens* (Willd.) Zizka | 20150192 | Yao, Wei | Huizhou Guangdong | MF063649 | MF064206 | MF065305 | MF064768 | MF065681 | MF073500 |
| *Melinis repens* (Willd.) Zizka | 20150365 | Yao, Wei | Nan’ao Guangdong | MF063650 | MF064207 | MF065306 | MF064769 | - | MF073501 |
| *Melinis repens* (Willd.) Zizka |  |  |  | AY746595 | GU134976 | - | KR735567 | KP878958 | - |
| *Melinis repens* (Willd.) Zizka |  |  |  | KP878894 | - | - | KR735624 | FJ486532 | - |
| *Melinis repens* (Willd.) Zizka |  |  |  | - | - | - | KR735779 | - | - |
| *Melinis repens* (Willd.) Zizka |  |  |  | - | - | - | KR735831 | - | - |
| *Miscanthus floridulus* (Lab.) Warb. ex Schum. et Laut. | 2014724 | Wei, Li,Zhang* | Pingtan Fujian | MF063613 | MF064166 | MF065272 | MF064729 | MF065642 | MF073502 |
| *Miscanthus floridulus* (Lab.) Warb. ex Schum. et Laut. | 20150533 | Yao, Wei | Wenzhou Zhejiang | MF063614 | MF064167 | MF065273 | MF064730 | MF065643 | MF073503 |
| *Miscanthus floridulus* (Lab.) Warb. ex Schum. et Laut. |  |  |  | AF345214 | - | - | - | - | - |
| *Miscanthus floridulus* (Lab.) Warb. ex Schum. et Laut. |  |  |  | AY116280 | - | - | - | - | - |
| *Miscanthus floridulus* (Lab.) Warb. ex Schum. et Laut. |  |  |  | AF345215 | - | - | - | - | - |
| *Miscanthus floridulus* (Lab.) Warb. ex Schum. et Laut. |  |  |  | AY116281 | - | - | - | - | - |
| *Miscanthus floridulus* (Lab.) Warb. ex Schum. et Laut. |  |  |  | EF211948 | - | - | - | - | - |
| *Miscanthus floridulus* (Lab.) Warb. ex Schum. et Laut. |  |  |  | EF211949 | - | - | - | - | - |
| *Miscanthus floridulus* (Lab.) Warb. ex Schum. et Laut. |  |  |  | JX156342 | - | - | - | - | - |
| *Miscanthus floridulus* (Lab.) Warb. ex Schum. et Laut. |  |  |  | KU297958 | - | - | - | - | - |
| *Miscanthus sinensis* Anderss. | 20141124 | Yao, Zhang | Fangchenggang Guangxi | MF063615 | MF064168 | - | MF064731 | MF065644 | MF073504 |
| *Miscanthus sinensis* Anderss. | 20141354 | Yao, Li, Wei | Wanning Hainan | - | MF064169 | - | MF064732 | MF065645 | MF073505 |
| *Miscanthus sinensis* Anderss. | 2014327 | Wei, Li, Zhang | Rongcheng Shandong | MF063616 | MF064170 | - | - | MF065646 | - |
| *Miscanthus sinensis* Anderss. | 20151245 | Yao, Wei, Yang | Qinghuangdao Hebei | - | - | - | - | - | MF073506 |
| *Miscanthus sinensis* Anderss. | 20151376 | Yao, Wei, Yang | Lianyungang Jiangsu | MF063617 | MF064171 | - | - | MF065647 | MF073507 |
| *Miscanthus sinensis* Anderss. |  |  |  | AF019822 | AM889726 | EF125118 | GQ248342 | - | EU434232 |
| *Miscanthus sinensis* Anderss. |  |  |  | AF345227 | EU434296 | KP711274 | HQ600094 | - | - |
| *Miscanthus sinensis* Anderss. |  |  |  | AF345228 | GQ248159 | - | KP711181 | - | - |
| *Miscanthus sinensis* Anderss. |  |  |  | AJ426565 | HG793999 | - | - | - | - |
| *Miscanthus sinensis* Anderss. |  |  |  | AJ426566 | - | - | - | - | - |
| *Miscanthus sinensis* Anderss. |  |  |  | AY116270 | - | - | - | - | - |
| *Miscanthus sinensis* Anderss. |  |  |  | AY116272 | - | - | - | - | - |
| *Miscanthus sinensis* Anderss. |  |  |  | AY116273 | - | - | - | - | - |
| *Miscanthus sinensis* Anderss. |  |  |  | AY116274 | - | - | - | - | - |
| *Miscanthus sinensis* Anderss. |  |  |  | AY116275 | - | - | - | - | - |
| *Miscanthus sinensis* Anderss. |  |  |  | AY116276 | - | - | - | - | - |
| *Miscanthus sinensis* Anderss. |  |  |  | EF211950 | - | - | - | - | - |
| *Miscanthus sinensis* Anderss. |  |  |  | EF211951 | - | - | - | - | - |
| *Miscanthus sinensis* Anderss. |  |  |  | HQ600498 | - | - | - | - | - |
| *Miscanthus sinensis* Anderss. |  |  |  | HQ822020 | - | - | - | - | - |
| *Miscanthus sinensis* Anderss. |  |  |  | HQ822021 | - | - | - | - | - |
| *Miscanthus sinensis* Anderss. |  |  |  | HQ822022 | - | - | - | - | - |
| *Miscanthus sinensis* Anderss. |  |  |  | HQ822023 | - | - | - | - | - |
| *Miscanthus sinensis* Anderss. |  |  |  | HQ822024 | - | - | - | - | - |
| *Miscanthus sinensis* Anderss. |  |  |  | HQ822025 | - | - | - | - | - |
| *Miscanthus sinensis* Anderss. |  |  |  | HQ822026 | - | - | - | - | - |
| *Miscanthus sinensis* Anderss. |  |  |  | JN544315 | - | - | - | - | - |
| *Miscanthus sinensis* Anderss. |  |  |  | JN544316 | - | - | - | - | - |
| *Miscanthus sinensis* Anderss. |  |  |  | JN544317 | - | - | - | - | - |
| *Miscanthus sinensis* Anderss. |  |  |  | JN544318 | - | - | - | - | - |
| *Miscanthus sinensis* Anderss. |  |  |  | JN544319 | - | - | - | - | - |
| *Miscanthus sinensis* Anderss. |  |  |  | JN544320 | - | - | - | - | - |
| *Miscanthus sinensis* Anderss. |  |  |  | JN544321 | - | - | - | - | - |
| *Miscanthus sinensis* Anderss. |  |  |  | JN544322 | - | - | - | - | - |
| *Miscanthus sinensis* Anderss. |  |  |  | JN544323 | - | - | - | - | - |
| *Miscanthus sinensis* Anderss. |  |  |  | JN544324 | - | - | - | - | - |
| *Miscanthus sinensis* Anderss. |  |  |  | JN544325 | - | - | - | - | - |
| *Miscanthus sinensis* Anderss. |  |  |  | KF163651 | - | - | - | - | - |
| *Miscanthus sinensis* Anderss. |  |  |  | KF163652 | - | - | - | - | - |
| *Miscanthus sinensis* Anderss. |  |  |  | KF163849 | - | - | - | - | - |
| *Miscanthus sinensis* Anderss. |  |  |  | KP711100 | - | - | - | - | - |
| *Miscanthus sinensis* Anderss. |  |  |  | KP711101 | - | - | - | - | - |
| *Miscanthus sinensis* Anderss. |  |  |  | KU297959 | - | - | - | - | - |
| *Miscanthus sinensis* Anderss. |  |  |  | KU297961 | - | - | - | - | - |
| *Miscanthus sinensis* Anderss. |  |  |  | KU297962 | - | - | - | - | - |
| *Miscanthus sinensis* Anderss. |  |  |  | KU297963 | - | - | - | - | - |
| *Miscanthus sinensis* Anderss. |  |  |  | KU297964 | - | - | - | - | - |
| *Neyraudia reynaudiana* (Kunth) Keng ex Hitchcock | 20150344 | Yao, Wei | Nan’ao Guangdong | MF063618 | MF064172 | MF065274 | MF064733 | MF065648 | - |
| *Neyraudia reynaudiana* (Kunth) Keng ex Hitchcock |  |  |  | - | GU135015 | EF125109 | GU135346.2 | GU359636 | - |
| *Neyraudia reynaudiana* (Kunth) Keng ex Hitchcock |  |  |  | - | JF729133 | HE575830 | - | HE575781 | - |
| *Panicum repens* L. | 20141014 | Yao, Zhang | Beihai Guangxi | MF063619 | MF064173 | MF065275 | MF064734 | MF065649 | MF073508 |
| *Panicum repens* L. | 20141114 | Yao, Zhang | Fangchenggang Guangxi | MF063620 | MF064174 | MF065276 | MF064735 | MF065650 | MF073509 |
| *Panicum repens* L. | 20141244 | Yao, Li, Wei | Wenchang Hainan | MF063621 | MF064175 | MF065277 | MF064736 | MF065651 | MF073510 |
| *Panicum repens* L. | 20141549 | Yao, Li, Wei | Dongfang Hainan | MF063622 | MF064176 | MF065278 | MF064737 | MF065652 | MF073511 |
| *Panicum repens* L. | 2014606 | Wei, Li,Zhang* | Ningde Fujian | MF063623 | MF064177 | MF065279 | MF064738 | MF065653 | MF073512 |
| *Panicum repens* L. | 2014819 | Wei, Li,Zhang* | Zhangzhou Fujian | MF063624 | MF064178 | MF065280 | MF064739 | MF065654 | MF073513 |
| *Panicum repens* L. | 20150093 | Yao, Wei | Zhanjiang Guangdong | MF063625 | MF064179 | MF065281 | MF064740 | MF065655 | MF073514 |
| *Panicum repens* L. | 20150251 | Yao, Wei | Huizhou Guangdong | MF063626 | MF064180 | MF065282 | MF064741 | MF065656 | MF073515 |
| *Panicum repens* L. |  |  |  | AY129722 | HM850546 | HE575855 | GU135308.2 | KM538710 | - |
| *Panicum repens* L. |  |  |  | HF934104 | - | HM850228 | - | U21993 | - |
| *Phacelurus latifolius* (Steud.) Ohwi | 20141656 | Yao, Li, Wei | Changjiang Hainan | MF063627 | MF064181 | - | MF064742 | MF065657 | MF073516 |
| *Phacelurus latifolius* (Steud.) Ohwi | 2014238 | Yao, Li, Wei | Cixi Zhejiang | MF063628 | MF064182 | - | MF064743 | MF065658 | MF073517 |
| *Phragmites australis* (Cav.) Trin. ex Steud. | 2014227 | Yao, Li, Wei | Cixi Zhejiang | MF063629 | MF064183 | MF065283 | MF064744 | MF065659 | MF073518 |
| *Phragmites australis* (Cav.) Trin. ex Steud. | 2014377 | Wei, Li, Zhang | Penglai Shandong | - | MF064184 | MF065284 | MF064745 | MF065660 | MF073519 |
| *Phragmites australis* (Cav.) Trin. ex Steud. | 2014485 | Wei, Li, Zhang | Dongying Shandong | - | MF064185 | MF065285 | MF064746 | MF065661 | MF073520 |
| *Phragmites australis* (Cav.) Trin. ex Steud. | 2014602 | Wei, Li,Zhang* | Ningde Fujian | - | MF064186 | MF065286 | MF064747 | MF065662 | MF073521 |
| *Phragmites australis* (Cav.) Trin. ex Steud. | 20160177 | Yao, Wei | Wulumuqi Xinjiang | - | MF064187 | - | MF064748 | MF065663 | - |
| *Phragmites australis* (Cav.) Trin. ex Steud. | 20160235 | Yao, Wei | Wulumuqi Xinjiang | - | MF064188 | - | MF064749 | MF065664 | MF073522 |
| *Phragmites australis* (Cav.) Trin. ex Steud. | 20160246 | Yao, Wei | Shihezi Xinjiang | - | - | - | MF064750 | - | MF073523 |
| *Phragmites australis* (Cav.) Trin. ex Steud. |  |  |  | AF019810 | JQ588790 | KF697233 | AY016310 | EF422913 | - |
| *Phragmites australis* (Cav.) Trin. ex Steud. |  |  |  | HQ600491 | JQ588791 | KF697234 | HQ596785 | U21997 | - |
| *Phragmites australis* (Cav.) Trin. ex Steud. |  |  |  | KP057062 | KJ593050 | KM360930 | HQ600088 | - | - |
| *Phragmites australis* (Cav.) Trin. ex Steud. |  |  |  | KP057063 | KJ593051 | U13229 | KC584966 | - | - |
| *Phragmites australis* (Cav.) Trin. ex Steud. |  |  |  | KP057064 | KJ772998 | - | - | - | - |
| *Phragmites australis* (Cav.) Trin. ex Steud. |  |  |  | KP057065 | - | - | - | - | - |
| *Phragmites australis* (Cav.) Trin. ex Steud. |  |  |  | KP711061 | - | - | - | - | - |
| *Poa acroleuca* Steud. | 20160266 | Yao, Wei | Shihezi Xinjiang | MF063635 | - | MF065289 | MF064752 | MF065667 | MF073526 |
| *Poa acroleuca* Steud. | 20150511 | Yao, Wei | Wenzhou Zhejiang | MF063630 | MF064189 | MF065287 | MF064751 | MF065665 | MF073524 |
| *Poa acroleuca* Steud. | 20150697 | Yao, Wei | Wenling Zhejiang | - | MF064190 | - | - | - | - |
| *Poa acroleuca* Steud. | 20150755 | Yao, Wei | Zhoushan Zhejiang | MF063631 | MF064191 | MF065288 | MF064753 | MF065666 | MF073525 |
| *Poa acroleuca* Steud. |  |  |  | HQ600547 | - | - | HQ600136 | KM538765 | - |
| *Poa prolixior* Rendle | 20160182 | Yao, Wei | Wulumuqi Xinjiang | MF063638 | MF064194 | MF065292 | MF064756 | MF065670 | MF073528 |
| *Poa prolixior* Rendle | 20160198 | Yao, Wei | Wulumuqi Xinjiang | - | MF064195 | MF065293 | MF064757 | - | MF073529 |
| *Poa prolixior* Rendle | 20150652 | Yao, Wei | Wenling Zhejiang | MF063636 | MF064192 | MF065290 | MF064754 | MF065668 | - |
| *Poa prolixior* Rendle | 20150730 | Yao, Wei | Yuhuan Zhejiang | MF063637 | MF064193 | MF065291 | MF064755 | MF065669 | MF073527 |
| *Polypogon fugax* Nees ex Steud. | 2014248 | Yao, Li, Wei | Cixi Zhejiang | MF063639 | MF064196 | MF065294 | MF064758 | MF065671 | MF073530 |
| *Polypogon fugax* Nees ex Steud. | 20150606 | Yao, Wei | Wenzhou Zhejiang | - | MF064197 | MF065295 | MF064759 | MF065672 | MF073531 |
| *Polypogon fugax* Nees ex Steud. | 20150610 | Yao, Wei | Wenling Zhejiang | MF063640 | - | MF065296 | - | MF065673 | MF073532 |
| *Polypogon fugax* Nees ex Steud. | 20150764 | Yao, Wei | Zhoushan Zhejiang | MF063641 | MF064198 | MF065297 | MF064760 | MF065674 | MF073533 |
| *Polypogon fugax* Nees ex Steud. | 20150821 | Yao, Wei | Zhoushan Zhejiang | MF063642 | MF064199 | MF065298 | MF064761 | MF065675 | MF073534 |
| *Polypogon fugax* Nees ex Steud. | 20150853 | Yao, Wei | Zhoushan Zhejiang | MF063643 | MF064200 | MF065299 | MF064762 | MF065676 | MF073535 |
| *Polypogon fugax* Nees ex Steud. | 20151440 | Yao, Wei, Yang | Yancheng Jiangsu | MF063644 | MF064201 | MF065300 | MF064763 | MF065677 | MF073536 |
| *Polypogon fugax* Nees ex Steud. |  |  |  | FJ821784 | KF713151 | KF713054 | HQ600160 | - | - |
| *Polypogon fugax* Nees ex Steud. |  |  |  | HQ600511 | KF713152 | KF713055 | - | - | - |
| *Polypogon fugax* Nees ex Steud. |  |  |  | KF713273 | KF713153 | KF713056 | - | - | - |
| *Polypogon fugax* Nees ex Steud. |  |  |  | KF713274 | KP135423 | KP135425 | - | - | - |
| *Polypogon fugax* Nees ex Steud. |  |  |  | KF713275 | KP135424 | KP135426 | - | - | - |
| *Polypogon fugax* Nees ex Steud. |  |  |  | KF713276 | - | - | - | - | - |
| *Polypogon fugax* Nees ex Steud. |  |  |  | KF713277 | - | - | - | - | - |
| *Polypogon fugax* Nees ex Steud. |  |  |  | KF713278 | - | - | - | - | - |
| *Polypogon fugax* Nees ex Steud. |  |  |  | KP135427 | - | - | - | - | - |
| *Polypogon fugax* Nees ex Steud. |  |  |  | KP135428 | - | - | - | - | - |
| *Rottboellia cochinchinensis* (Loureiro) Clayton | 20141105 | Yao, Zhang | Fangchenggang Guangxi | MF063651 | MF064208 | MF065307 | MF064770 | MF065682 | MF073537 |
| *Rottboellia cochinchinensis* (Loureiro) Clayton | 2014821 | Wei, Li,Zhang* | Zhangzhou Fujian | MF063652 | MF064209 | MF065308 | MF064771 | MF065683 | MF073538 |
| *Saccharum arundinaceum* Retz. | 20141359 | Yao, Li, Wei | Wanning Hainan | MF063653 | MF064210 | - | MF064772 | MF065684 | MF073539 |
| *Saccharum arundinaceum* Retz. | 20141450 | Yao, Li, Wei | Lingshui Hainan | MF063654 | MF064211 | MF065309 | MF064773 | - | MF073540 |
| *Saccharum arundinaceum* Retz. | 20150092 | Yao, Wei | Zhanjiang Guangdong | MF063655 | MF064212 | MF065310 | MF064774 | MF065685 | MF073541 |
| *Saccharum arundinaceum* Retz. |  |  |  | AB281160 | - | - | - | - | - |
| *Saccharum arundinaceum* Retz. |  |  |  | EF211956 | - | - | - | - | - |
| *Saccharum arundinaceum* Retz. |  |  |  | GQ870229 | - | - | - | - | - |
| *Saccharum arundinaceum* Retz. |  |  |  | JX156345 | - | - | - | - | - |
| *Setaria faberii* Herrm. | 2014597 | Wei, Li,Zhang* | Ningde Fujian | MF063632 | MF064213 | MF065311 | - | MF065686 | MF073542 |
| *Setaria faberii* Herrm. | 2014628 | Wei, Li,Zhang* | Ningde Fujian | MF063633 | MF064214 | MF065312 | MF064775 | MF065687 | MF073543 |
| *Setaria viridis* (L.) Beauv. | 20141239 | Yao, Wei, Yang | Qinghuangdao Hebei | MF063656 | MF064215 | MF065313 | MF064776 | MF065688 | MF073544 |
| *Setaria viridis* (L.) Beauv. | 2014231 | Yao, Li, Wei | Cixi Zhejiang | MF063657 | MF064216 | MF065314 | MF064777 | MF065689 | MF073545 |
| *Setaria viridis* (L.) Beauv. | 2014315 | Wei, Li, Zhang | Rongcheng Shandong | MF063658 | MF064217 | MF065315 | MF064778 | MF065690 | MF073546 |
| *Setaria viridis* (L.) Beauv. | 2014399 | Wei, Li, Zhang | Penglai Shandong | MF063659 | MF064218 | MF065316 | MF064779 | MF065691 | MF073547 |
| *Setaria viridis* (L.) Beauv. | 2014716 | Wei, Li,Zhang* | Pingtan Fujian | MF063660 | MF064219 | MF065317 | MF064780 | MF065692 | MF073548 |
| *Setaria viridis* (L.) Beauv. | 20160179 | Yao, Wei | Wulumuqi Xinjiang | MF063661 | MF064220 | MF065318 | MF064781 | - | MF073550 |
| *Setaria viridis* (L.) Beauv. | 20160244 | Yao, Wei | Shihezi Xinjiang | MF063662 | MF064221 | - | MF064782 | - | MF073549 |
| *Setaria viridis* (L.) Beauv. | 20160259 | Yao, Wei | Shihezi Xinjiang | MF063663 | MF064222 | MF065319 | MF064783 | - | - |
| *Setaria viridis* (L.) Beauv. |  |  |  | FJ766179 | - | - | - | - | - |
| *Setaria viridis* (L.) Beauv. |  |  |  | HQ600484 | - | - | - | - | - |
| *Setaria viridis* (L.) Beauv. |  |  |  | HQ600485 | - | - | - | - | - |
| *Setaria viridis* (L.) Beauv. |  |  |  | KF163676 | - | - | - | - | - |
| *Setaria viridis* (L.) Beauv. |  |  |  | KF163677 | - | - | - | - | - |
| *Setaria viridis* (L.) Beauv. |  |  |  | KF163678 | - | - | - | - | - |
| *Setaria viridis* (L.) Beauv. |  |  |  | KF163679 | - | - | - | - | - |
| *Setaria viridis* (L.) Beauv. |  |  |  | KF163680 | - | - | - | - | - |
| *Setaria viridis* (L.) Beauv. |  |  |  | KF163681 | - | - | - | - | - |
| *Setaria viridis* (L.) Beauv. |  |  |  | KF954527 | - | - | - | - | - |
| *Setaria viridis* (L.) Beauv. |  |  |  | KX147546 | - | - | - | - | - |
| *Spartina alterniflora* Lois. | 2014240 | Yao, Li, Wei | Cixi Zhejiang | MF063664 | MF064223 | - | - | MF065693 | MF073551 |
| *Spartina alterniflora* Lois. | 2014514 | Wei, Li, Zhang | Dongying Shandong | - | MF064224 | MF065320 | MF064784 | MF065694 | MF073552 |
| *Spartina alterniflora* Lois. | 2014580 | Wei, Li,Zhang* | Ningde Fujian | MF063665 | MF064225 | - | MF064785 | MF065695 | MF073553 |
| *Spartina alterniflora* Lois. | 2014601 | Wei, Li,Zhang* | Ningde Fujian | MF063666 | MF064226 | - | - | MF065696 | MF073554 |
| *Spartina alterniflora* Lois. | 2014749 | Wei, Li,Zhang* | Pingtan Fujian | MF063667 | MF064227 | - | MF064786 | MF065697 | MF073555 |
| *Spartina alterniflora* Lois. | 2014805 | Wei, Li,Zhang* | Zhangzhou Fujian | MF063668 | MF064228 | MF065321 | - | MF065698 | MF073556 |
| *Spartina alterniflora* Lois. | 20150025 | Yao, Wei | Zhanjiang Guangdong | - | MF064229 | MF065322 | MF064787 | MF065699 | MF073557 |
| *Spartina alterniflora* Lois. | 201505077 | Liu | Chongming Shanghai | MF063669 | MF064230 | MF065323 | MF064788 | MF065700 | MF073558 |
| *Spartina alterniflora* Lois. | 20150622 | Yao, Wei | Wenling Zhejiang | MF063670 | MF064231 | MF065324 | MF064789 | MF065701 | MF073559 |
| *Spartina alterniflora* Lois. | 20150835 | Yao, Wei | Zhoushan Zhejiang | - | MF064232 | MF065325 | MF064790 | MF065702 | MF073560 |
| *Spartina alterniflora* Lois. |  |  |  | AF272775 | - | - | - | - | - |
| *Spartina alterniflora* Lois. |  |  |  | AJ489594 | - | - | - | - | - |
| *Spartina alterniflora* Lois. |  |  |  | AJ489595 | - | - | - | - | - |
| *Spartina alterniflora* Lois. |  |  |  | AJ489596 | - | - | - | - | - |
| *Spartina alterniflora* Lois. |  |  |  | AJ489597 | - | - | - | - | - |
| *Spartina alterniflora* Lois. |  |  |  | AJ489598 | - | - | - | - | - |
| *Spartina alterniflora* Lois. |  |  |  | JX852435 | - | - | - | - | - |
| *Spartina alterniflora* Lois. |  |  |  | JX852436 | - | - | - | - | - |
| *Spartina alterniflora* Lois. |  |  |  | JX852437 | - | - | - | - | - |
| *Spartina alterniflora* Lois. |  |  |  | KM010330 | - | - | - | - | - |
| *Spartina alterniflora* Lois. |  |  |  | KM010331 | - | - | - | - | - |
| *Spinifex littoreus* (Burm. f. ) Merr. | 20141010 | Yao, Zhang | Beihai Guangxi | MF063671 | MF064233 | MF065326 | MF064791 | MF065703 | MF073561 |
| *Spinifex littoreus* (Burm. f. ) Merr. | 20141301 | Yao, Li, Wei | Wanning Hainan | MF063672 | MF064234 | MF065327 | MF064792 | MF065704 | MF073562 |
| *Spinifex littoreus* (Burm. f. ) Merr. | 20141462 | Yao, Li, Wei | Lingshui Hainan | MF063673 | MF064235 | MF065328 | - | MF065705 | MF073563 |
| *Spinifex littoreus* (Burm. f. ) Merr. | 2014860 | Wei, Li,Zhang* | Zhangzhou Fujian | MF063674 | MF064236 | - | MF064793 | MF065706 | MF073564 |
| *Spinifex littoreus* (Burm. f. ) Merr. |  |  |  | GQ870180 | - | - | - | - | - |
| *Sporobolus diander* (Retz.) Beauv. | 20141408 | Yao, Li, Wei | Wanning Hainan | MF063675 | - | MF065329 | MF064794 | - | MF073565 |
| *Sporobolus diander* (Retz.) Beauv. | 2014702 | Wei, Li,Zhang* | Pingtan Fujian | MF063676 | MF064237 | MF065330 | MF064795 | - | MF073566 |
| *Sporobolus diander* (Retz.) Beauv. | 20150047 | Yao, Wei | Zhanjiang Guangdong | MF063677 | MF064238 | MF065331 | MF064796 | - | MF073567 |
| *Sporobolus diander* (Retz.) Beauv. | 20150281 | Yao, Wei | Huizhou Guangdong | MF063678 | MF064239 | MF065332 | MF064797 | - | MF073568 |
| *Sporobolus diander* (Retz.) Beauv. | 20150363 | Yao, Wei | Nan’ao Guangdong | MF063679 | - | MF065333 | MF064798 | - | MF073569 |
| *Sporobolus fertilis* (Steud.) W. D. Clayt. | 2014592 | Wei, Li,Zhang* | Ningde Fujian | MF063680 | MF064240 | MF065334 | MF064799 | - | MF073570 |
| *Sporobolus fertilis* (Steud.) W. D. Clayt. | 20150411 | Yao, Wei | Nan’ao Guangdong | MF063681 | MF064241 | MF065335 | MF064800 | - | MF073571 |
| *Sporobolus fertilis* (Steud.) W. D. Clayt. | 20150589 | Yao, Wei | Wenzhou Zhejiang | MF063682 | - | - | MF064801 | - | MF073572 |
| *Sporobolus fertilis* (Steud.) W. D. Clayt. |  |  |  | EU646103 | - | - | - | - | - |
| *Sporobolus fertilis* (Steud.) W. D. Clayt. |  |  |  | EU646104 | - | - | - | - | - |
| *Sporobolus fertilis* (Steud.) W. D. Clayt. |  |  |  | EU646105 | - | - | - | - | - |
| *Sporobolus fertilis* (Steud.) W. D. Clayt. |  |  |  | KM010413 | - | - | - | - | - |
| *Sporobolus fertilis* (Steud.) W. D. Clayt. |  |  |  | KM010414 | - | - | - | - | - |
| *Sporobolus fertilis* (Steud.) W. D. Clayt. |  |  |  | KP057070 | - | - | - | - | - |
| *Sporobolus fertilis* (Steud.) W. D. Clayt. |  |  |  | KP057071 | - | - | - | - | - |
| *Sporobolus fertilis* (Steud.) W. D. Clayt. |  |  |  | KP057072 | - | - | - | - | - |
| *Sporobolus fertilis* (Steud.) W. D. Clayt. |  |  |  | KP057073 | - | - | - | - | - |
| *Sporobolus fertilis* (Steud.) W. D. Clayt. |  |  |  | KP057074 | - | - | - | - | - |
| *Sporobolus fertilis* (Steud.) W. D. Clayt. |  |  |  | KP057075 | - | - | - | - | - |
| *Sporobolus fertilis* (Steud.) W. D. Clayt. |  |  |  | KP057076 | - | - | - | - | - |
| *Sporobolus fertilis* (Steud.) W. D. Clayt. |  |  |  | KP711110 | - | - | - | - | - |
| *Sporobolus virginicus* (L.) Kunth | 20141451 | Yao, Li, Wei | Lingshui Hainan | MF063683 | MF064242 | MF065336 | MF064802 | - | MF073573 |
| *Sporobolus virginicus* (L.) Kunth | 20141568 | Yao, Li, Wei | Dongfang Hainan | MF063684 | MF064243 | MF065337 | MF064803 | - | MF073574 |
| *Sporobolus virginicus* (L.) Kunth | 2014639 | Wei, Li,Zhang* | Ningde Fujian | - | MF064244 | - | MF064804 | - | MF073575 |
| *Sporobolus virginicus* (L.) Kunth | 2014807 | Wei, Li,Zhang* | Zhangzhou Fujian | - | MF064245 | MF065338 | MF064805 | - | MF073576 |
| *Sporobolus virginicus* (L.) Kunth | 20150074 | Yao, Wei | Zhanjiang Guangdong | - | MF064246 | - | MF064806 | - | MF073577 |
| *Sporobolus virginicus* (L.) Kunth |  |  |  | EU646114 | - | - | - | - | - |
| *Sporobolus virginicus* (L.) Kunth |  |  |  | EU646115 | - | - | - | - | - |
| *Sporobolus virginicus* (L.) Kunth |  |  |  | EU646116 | - | - | - | - | - |
| *Sporobolus virginicus* (L.) Kunth |  |  |  | KM010407 | - | - | - | - | - |
| *Zoysia matrella* (L. ) Merr. | 20150054 | Yao, Wei | Zhanjiang Guangdong | MF063685 | MF064247 | MF065339 | MF064807 | MF065707 | MF073578 |
| *Zoysia matrella* (L. ) Merr. | 20150680 | Yao, Wei | Wenling Zhejiang | MF063686 | MF064248 | MF065340 | MF064808 | MF065708 | MF073579 |
| *Zoysia matrella* (L. ) Merr. |  |  |  | EF153099 | - | - | - | - | - |
| *Zoysia matrella* (L. ) Merr. |  |  |  | GQ478117 | - | - | - | - | - |
| *Zoysia sinica* Hance | 20141283 | Yao, Li, Wei | Wenchang Hainan | - | MF064249 | MF065341 | - | MF065709 | MF073580 |
| *Zoysia sinica* Hance | 20150215 | Yao, Wei | Huizhou Guangdong | MF063687 | MF064250 | MF065342 | MF064809 | MF065710 | MF073581 |
| *Zoysia sinica* Hance | 20150451 | Yao, Wei | Nan’ao Guangdong | MF063688 | MF064251 | MF065343 | MF064810 | MF065711 | MF073582 |
| *Zoysia sinica* Hance | 20150542 | Yao, Wei | Wenzhou Zhejiang | MF063689 | MF064252 | MF065344 | MF064811 | MF065712 | MF073583 |
| *Zoysia sinica* Hance | 20150710 | Yao, Wei | Wenling Zhejiang | MF063690 | MF064253 | MF065345 | MF064812 | MF065713 | MF073584 |
| *Zoysia sinica* Hance |  |  |  | HQ600482 | - | - | - | - | - |
| *Zoysia sinica* Hance |  |  |  | KP057080 | - | - | - | - | - |
| *Zoysia sinica* Hance |  |  |  | KP057081 | - | - | - | - | - |
| *Zoysia sinica* Hance |  |  |  | KP057082 | - | - | - | - | - |
| *Zoysia sinica* Hance |  |  |  | KP057083 | - | - | - | - | - |
| *Zoysia sinica* Hance |  |  |  | KP057084 | - | - | - | - | - |
| *Zoysia sinica* Hance |  |  |  | KP711112 | - | - | - | - | - |

Note： Li：Hong-Qing Li; Liu：Wen-liang Liu; Wei：Ya-Nan Wei; Yang：Zhu-Ai Yang; Yao：Peng-Cheng Yao; Zhang：Zhen Zhang; Zhang*：Li-Fang Zhang.

Accession numbers begin with “MF” are newly generated in this paper.
