# Supplementary material for: Evaluating sampling strategy for DNA barcoding study of coastal and inland halo-tolerant Poaceae and Chenopodiaceae: A case study for increased sample size
Source: PLoS One. 2017 Sep 21;12(9):e0185311. doi: 10.1371/journal.pone.0185311 (PMC5608404; doi:10.1371/journal.pone.0185311)
Supplement: S2 Table — (DOCX) [file pone.0185311.s004.docx]

**TableS2** Details of Chenopodiaceae material included in this study

| Taxon | Voucher Numbers | Collecter | Locality | GenBank accession | | | | | |
| --- | --- | --- | --- | --- | --- | --- | --- | --- | --- |
|  |  |  |  | *ITS* | *ma*tK | *rbc*L | *trn*H-*psb*A | *trn*L-F | *atp*B*-rbc*L |
| *Atriplex maximowicziana* Makino | 2014802 | Wei, Li, Zhang* | Zhangzhou Fujian | MF063349 | MF063884 | MF064997 | MF064446 | MF073585 | MF073743 |
| *Atriplex maximowicziana* Makino | 2014842 | Wei, Li, Zhang* | Zhangzhou Fujian | MF063350 | MF063885 | MF064998 | MF064447 | MF073586 | MF073744 |
| *Atriplex maximowicziana* Makino | 20141534 | Yao, Li, Wei | Dongfang Hainan | MF063351 | MF063886 | MF064999 | MF064448 | MF073587 | MF073745 |
| *Atriplex maximowicziana* Makino | 20150453 | Yao, Wei | Nan’ao Guangdong | MF063352 | MF063887 | MF065000 | MF064449 | MF073588 | MF073746 |
| *Atriplex maximowicziana* Makino | 20141630 | Yao, Li, Wei | Changjiang Hainan | MF063353 | MF063888 | MF065001 | MF064450 | MF073589 | MF073747 |
| *Atriplex maximowicziana* Makino | 20150013 | Yao, Wei | Zhanjiang Guangdong | MF063354 | MF063889 | MF065002 | MF064451 | MF073590 | MF073748 |
| *Atriplex patens* (Litv.) Iljin | 2014323 | Wei, Li, Zhang | Rongcheng Shandong | MF063355 | MF063890 | MF065003 | - | MF073591 | MF073749 |
| *Atriplex patens* (Litv.) Iljin | 20151061 | Yao, Wei | Zhanjiang Guangdong | MF063356 | MF063891 | MF065004 | MF064452 | MF073592 | MF073750 |
| *Atriplex patens* (Litv.) Iljin | 20151087 | Yao, Li, Wei | Dalian Liaoning | MF063357 | MF063892 | MF065005 | MF064453 | MF073593 | MF073751 |
| *Atriplex patens* (Litv.) Iljin | 20151217 | Yao, Li, Wei | Huludao Liaoning | MF063358 | MF063893 | MF065006 | MF064454 | MF073594 | MF073752 |
| *Atriplex patens* (Litv.) Iljin | 20151291 | Yao, Wei, Yang | Qinghuangdao Hebei | MF063359 | MF063894 | MF065007 | MF064455 | MF073595 | MF073753 |
| *Atriplex patens* (Litv.) Iljin | 20151339 | Yao, Wei, Yang | Lianyungang Jiangsu | MF063360 | MF063895 | MF065008 | MF064456 | MF073596 | MF073754 |
| *Chenopodium acuminatum* Willd. | 20150383 | Yao, Wei | Nan’ao Guangdong | MF063361 | MF063896 | MF065009 | MF064457 | MF073597 | MF073755 |
| *Chenopodium acuminatum* Willd. | 20150132 | Yao, Wei | Zhanjiang Guangdong | MF063362 | MF063897 | MF065010 | MF064458 | MF073598 | MF073756 |
| *Chenopodium acuminatum* Willd. | 20150189 | Yao, Wei | Huidong Guangdong | MF063363 | MF063898 | MF065011 | MF064459 | MF073599 | MF073757 |
| *Chenopodium acuminatum* Willd. | 20151304 | Yao, Wei, Yang | Qinghuangdao Hebei | MF063364 | MF063899 | MF065012 | MF064460 | MF073600 | MF073758 |
| *Chenopodium acuminatum* Willd. | 2014685 | Wei, Li, Zhang* | Pingtan Fujian | MF063365 | MF063884 | MF065013 | MF064461 | MF073601 | MF073759 |
| *Chenopodium acuminatum* Willd. | 2014699 | Wei, Li, Zhang* | Pingtan Fujian | MF063366 | MF063885 | MF065014 | MF064462 | MF073602 | MF073760 |
| *Chenopodium acuminatum* Willd. |  |  |  | KU359316 | - | AY270077 | - | - | HM587668 |
| *Chenopodium album* L. | 2014177 | Li, Zhang | Chongming Shanghai | MF063367 | - | MF065015 | MF064463 | MF073603 | MF073761 |
| *Chenopodium album* L. | 2014184 | Li, Zhang | Chongming Shanghai | MF063368 | MF063900 | MF065016 | MF064464 | MF073604 | MF073762 |
| *Chenopodium album* L. | 2014245 | Yao, Li, Wei | Cixi Zhejiang | MF063369 | MF063901 | MF065017 | MF064465 | MF073605 | MF073763 |
| *Chenopodium album* L. | 2014314 | Wei, Li, Zhang | Rongcheng Shandong | MF063370 | MF063902 | MF065018 | MF064466 | MF073606 | MF073764 |
| *Chenopodium album* L. | 2014398 | Wei, Li, Zhang | Penglai Shandong | MF063371 | MF063903 | MF065019 | MF064467 | MF073607 | MF073765 |
| *Chenopodium album* L. | 2014489 | Wei, Li, Zhang | Dongying Shandong | MF063372 | MF063904 | MF065020 | MF064468 | MF073608 | MF073766 |
| *Chenopodium album* L. | 2014577 | Wei, Li, Zhang* | Ningde Fujian | MF063373 | MF063905 | MF065021 | MF064469 | MF073609 | MF073767 |
| *Chenopodium album* L. | 2014649 | Wei, Li, Zhang* | Ningde Fujian | MF063374 | MF063906 | - | MF064470 | MF073610 | MF073768 |
| *Chenopodium album* L. | 2014689 | Wei, Li, Zhang* | Pingtan Fujian | MF063375 | MF063907 | MF065022 | MF064471 | MF073611 | MF073769 |
| *Chenopodium album* L. | 2014708 | Wei, Li, Zhang* | Pingtan Fujian | MF063376 | MF063908 | MF065043 | MF064472 | MF073612 | MF073770 |
| *Chenopodium album* L. | 2014779 | Wei, Li, Zhang* | Zhangzhou Fujian | MF063377 | MF063909 | MF065023 | MF064473 | MF073613 | MF073771 |
| *Chenopodium album* L. | 20150168 | Yao, Wei | Zhanjiang Guangdong | MF063378 | MF063910 | MF065024 | MF064474 | MF073614 | MF073772 |
| *Chenopodium album* L. | 20150500 | Yao, Wei | Wenzhou Zhejiang | MF063379 | MF063911 | MF065025 | MF064475 | MF073615 | MF073773 |
| *Chenopodium album* L. | 20150696 | Yao, Wei | Wenling Zhejiang | MF063380 | MF063912 | MF065026 | MF064476 | MF073616 | MF073774 |
| *Chenopodium album* L. | 20150700 | Yao, Wei | Wenling Zhejiang | MF063381 | MF063913 | MF065027 | MF064477 | MF073617 | MF073775 |
| *Chenopodium album* L. | 20150714 | Yao, Wei | Yuhuan Zhejiang | MF063382 | MF063914 | MF065028 | MF064478 | MF073618 | MF073776 |
| *Chenopodium album* L. | 20150745 | Yao, Wei | Zhoushan Zhejiang | MF063383 | MF063915 | MF065029 | MF064479 | MF073619 | MF073777 |
| *Chenopodium album* L. | 20151000 | Yao, Li, Wei | Dalian Liaoning | MF063384 | MF063916 | - | MF064480 | MF073620 | MF073778 |
| *Chenopodium album* L. | 20151040 | Yao, Li, Wei | Dalian Liaoning | MF063385 | MF063917 | MF065030 | MF064481 | MF073621 | MF073779 |
| *Chenopodium album* L. | 20151042 | Yao, Li, Wei | Dalian Liaoning | MF063386 | MF063918 | MF065031 | MF064482 | MF073622 | MF073780 |
| *Chenopodium album* L. | 20151043 | Yao, Li, Wei | Dalian Liaoning | MF063387 | MF063919 | MF065032 | MF064483 | MF073623 | MF073781 |
| *Chenopodium album* L. | 20151064 | Yao, Li, Wei | Dalian Liaoning | MF063388 | MF063920 | MF065033 | MF064484 | MF073624 | MF073782 |
| *Chenopodium album* L. | 20151163 | Yao, Li, Wei | Huludao Liaoning | MF063389 | MF063921 | MF065034 | MF064485 | MF073625 | MF073783 |
| *Chenopodium album* L. | 20151167 | Yao, Li, Wei | Huludao Liaoning | MF063390 | MF063922 | MF065035 | MF064486 | MF073626 | MF073784 |
| *Chenopodium album* L. | 20151237 | Yao, Wei, Yang | Qinghuangdao Hebei | MF063391 | MF063923 | MF065036 | MF064487 | MF073627 | MF073785 |
| *Chenopodium album* L. | 20151267 | Yao, Wei, Yang | Qinghuangdao Hebei | MF063392 | MF063924 | MF065037 | MF064488 | MF073628 | MF073786 |
| *Chenopodium album* L. | 20151310 | Yao, Wei, Yang | Lianyungang Jiangsu | MF063393 | MF063925 | MF065038 | MF064489 | MF073629 | MF073787 |
| *Chenopodium album* L. | 20151322 | Yao, Wei, Yang | Lianyungang Jiangsu | MF063394 | MF063926 | MF065039 | MF064490 | MF073630 | MF073788 |
| *Chenopodium album* L. | 20151396 | Yao, Wei, Yang | Yancheng Jiangsu | MF063395 | MF063927 | MF065040 | MF064491 | MF073631 | MF073789 |
| *Chenopodium album* L. | 20151417 | Yao, Wei, Yang | Yancheng Jiangsu | MF063396 | MF063928 | MF065041 | MF064492 | MF073632 | MF073790 |
| *Chenopodium album* L. | 20151429 | Yao, Wei, Yang | Yancheng Jiangsu | MF063397 | MF063929 | MF065042 | MF064493 | MF073633 | MF073791 |
| *Chenopodium album* L. | 20160180 | Yao,Wei | Wulumuqi Xinjiang | MF063398 | MF063930 | MF065044 | MF064494 | MF073634 | MF073792 |
| *Chenopodium album* L. | 20160192 | Yao,Wei | Wulumuqi Xinjiang | MF063399 | MF063931 | MF065045 | MF064495 | MF073635 | MF073793 |
| *Chenopodium album* L. | 20160206 | Yao,Wei | Wulumuqi Xinjiang | MF063400 | MF063932 | MF065046 | MF064496 | MF073636 | MF073794 |
| *Chenopodium album* L. | 20160245 | Yao,Wei | Shihezi Xinjiang | MF063401 | MF063933 | MF065047 | MF064497 | MF073637 | - |
| *Chenopodium album* L. |  |  |  | FN561545 | HE855644 | KF319012 | JN044292 | HE577596 | - |
| *Chenopodium album* L. |  |  |  | FN561547 | HE855664 | KF319013 | JN044291 | HE577593 | - |
| *Chenopodium album* L. |  |  |  | FN561549 | HE855665 | KF319014 | JN044290 | HE577570 | - |
| *Chenopodium album* L. |  |  |  | FN561550 | HM850763 | KF319015 | JN044289 | HE577568 | - |
| *Chenopodium album* L. |  |  |  | HE577414 | HQ593232 | - | JN044288 | - | - |
| *Chenopodium album* L. |  |  |  | HE577418 | JF953529 | - | JN044287 | - | - |
| *Chenopodium album* L. |  |  |  | HE577419 | JF953530 | - | JN044286 | - | - |
| *Chenopodium album* L. |  |  |  | HE577420 | JF953531 | - | JN044284 | - | - |
| *Chenopodium album* L. |  |  |  | HE577421 | JF953532 | - | JN044282 | - | - |
| *Chenopodium album* L. |  |  |  | HE577430 | JF953533 | - | JN044281 | - | - |
| *Chenopodium album* L. |  |  |  | HE577432 | JF953534 | - | JN044279 | - | - |
| *Chenopodium album* L. |  |  |  | HE577453 | JF953536 | - | JN044278 | - | - |
| *Chenopodium album* L. |  |  |  | HE577456 | JF953537 | - | JN044277 | - | - |
| *Chenopodium album* L. |  |  |  | HE577457 | JF953539 | - | JN044276 | - | - |
| *Chenopodium album* L. |  |  |  | KU359317 | JF953541 | - | JN044275 | - | - |
| *Chenopodium album* L. |  |  |  | KF954518 | JF953542 | - | JN044274 | - | - |
| *Chenopodium album* L. |  |  |  | KP226639 | JF953543 | - | JN044273 | - | - |
| *Chenopodium album* L. |  |  |  | KP226640 | JF953544 | - | KM224552 | - | - |
| *Chenopodium album* L. |  |  |  | KP226641 | JF953545 | - | KF954533 | - | - |
| *Chenopodium album* L. |  |  |  | KP226638 | JF953546 | - | - | - | - |
| *Chenopodium album* L. |  |  |  | - | JF953547 | - | - | - | - |
| *Chenopodium album* L. |  |  |  | - | JN894388 | - | - | - | - |
| *Chenopodium album* L. |  |  |  | - | JN894520 | - | - | - | - |
| *Chenopodium album* L. |  |  |  | - | JN895260 | - | - | - | - |
| *Chenopodium serotinum* L. | 20150015 | Yao, Wei | Zhanjiang Guangdong | MF063413 | MF063947 | MF065061 | MF064511 | MF073651 | MF073808 |
| *Chenopodium serotinum* L. | 20150181 | Yao, Wei | Huizhou Guangdong | MF063414 | MF063948 | MF065062 | MF064512 | MF073652 | MF073809 |
| *Chenopodium serotinum* L. | 20150618 | Yao, Wei | Yancheng Jiangsu | MF063415 | MF063949 | MF065063 | MF064513 | MF073653 | MF073810 |
| *Chenopodium serotinum* L. | 20151421 | Yao, Wei, Yang | Yancheng Jiangsu | MF063416 | MF063950 | MF065064 | MF064514 | MF073654 | MF073811 |
| *Chenopodium serotinum* L. | 2015268 | Wei, Zhang | Fengxian Shanghai | MF063417 | MF063951 | MF065065 | MF064515 | MF073655 | MF073812 |
| *Chenopodium glaucum* L. | 2014244 | Yao, Li, Wei | Cixi Zhejiang | MF063402 | MF063934 | MF065048 | MF064498 | MF073638 | MF073795 |
| *Chenopodium glaucum* L. | 2014263 | Wei, Zhang | Fengxian Shanghai | MF063403 | MF063935 | MF065049 | MF064499 | MF073639 | MF073796 |
| *Chenopodium glaucum* L. | 2014455 | Wei, Li, Zhang | Penglai Shandong | MF063404 | MF063936 | MF065050 | MF064500 | MF073640 | MF073797 |
| *Chenopodium glaucum* L. | 2014499 | Wei, Li, Zhang | Dongying Shandong | MF063405 | MF063937 | MF065051 | MF064501 | MF073641 | MF073798 |
| *Chenopodium glaucum* L. | 2014729 | Wei, Li, Zhang* | Pingtan Fujian | MF063406 | MF063938 | MF065052 | MF064502 | MF073642 | MF073799 |
| *Chenopodium glaucum* L. | 20150614 | Yao, Wei | Wenling Zhejiang | MF063407 | MF063939 | MF065053 | MF064503 | MF073643 | MF073800 |
| *Chenopodium glaucum* L. | 20150839 | Yao, Wei | Zhoushan Zhejiang | - | MF063940 | MF065054 | MF064504 | MF073644 | MF073801 |
| *Chenopodium glaucum* L. | 20151132 | Yao, Li, Wei | Dalian Liaoning | MF063408 | MF063941 | MF065055 | MF064505 | MF073645 | MF073802 |
| *Chenopodium glaucum* L. | 20151294 | Yao, Wei, Yang | Qinghuangdao Hebei | MF063409 | MF063942 | MF065056 | MF064506 | MF073646 | MF073803 |
| *Chenopodium glaucum* L. | 20151343 | Yao, Wei, Yang | Lianyungang Jiangsu | MF063410 | MF063943 | MF065057 | MF064507 | MF073647 | MF073804 |
| *Chenopodium glaucum* L. | 20151420 | Yao, Wei, Yang | Yancheng Jiangsu | MF063411 | MF063944 | MF065058 | MF064508 | MF073648 | MF073805 |
| *Chenopodium glaucum* L. | 20160215 | Yao, Wei | Wulumuqi Xinjiang | - | MF063945 | MF065059 | MF064509 | MF073649 | MF073806 |
| *Chenopodium glaucum* L. | 20160240 | Yao, Wei | Tulufan Xinjiang | MF063412 | MF063946 | MF065060 | MF064510 | MF073650 | MF073807 |
| *Chenopodium glaucum* L. |  |  |  | HE577386 | JF953551 | JF941273 | - | HE577526 | - |
| *Chenopodium glaucum* L. |  |  |  | HE577387 | JF953552 | JF941274 | - | HE577527 | - |
| *Chenopodium glaucum* L. |  |  |  | JF976149 | JF953550 | JF941275 | - | - | - |
| *Chenopodium glaucum* L. |  |  |  | JF976150 | JF953553 | JF941276 | - | - | - |
| *Chenopodium glaucum* L. |  |  |  | JF976151 | - | - | - | - | - |
| *Chenopodium glaucum* L. |  |  |  | JF976152 | - | - | - | - | - |
| *Corispermum puberulum* Iljin | 2014371 | Wei, Li, Zhang | Penglai Shandong | MF063418 | MF063952 | MF065066 | MF064516 | MF073656 | MF073813 |
| *Corispermum puberulum* Iljin | 20151071 | Yao, Li, Wei | Dalian Liaoning | MF063419 | MF063953 | MF065067 | MF064517 | MF073657 | MF073814 |
| *Corispermum puberulum* Iljin | 20151211 | Yao, Li, Wei | Huludao Liaoning | MF063420 | MF063954 | MF065068 | MF064518 | MF073658 | MF073815 |
| *Corispermum puberulum* Iljin | 20151227 | Yao, Li, Wei | Huludao Liaoning | MF063421 | MF063955 | MF065069 | MF064519 | MF073659 | MF073816 |
| *Corispermum puberulum* Iljin | 20151242 | Yao, Wei, Yang | Qinghuangdao Hebei | MF063422 | MF063956 | MF065070 | MF064520 | MF073660 | MF073817 |
| *Corispermum puberulum* Iljin | 20151302 | Yao, Wei, Yang | Qinghuangdao Hebei | MF063423 | MF063957 | MF065071 | MF064521 | MF073661 | MF073818 |
| *Corispermum puberulum* Iljin |  |  |  | JF792749 | - | JF792799 | - | - | - |
| *Corispermum puberulum* Iljin |  |  |  | JF792750 | - | JF792800 | - | - | - |
| *Dysphania ambrosioides* (L.) Mosyakin et Clemants | 2014576 | Wei, Li, Zhang* | Ningde Fujian | MF063424 | MF063958 | MF065072 | MF064523 | MF073662 | MF073819 |
| *Dysphania ambrosioides* (L.) Mosyakin et Clemants | 2014661 | Wei, Li, Zhang* | Ningde Fujian | MF063425 | MF063959 | MF065073 | MF064524 | MF073663 | MF073820 |
| *Dysphania ambrosioides* (L.) Mosyakin et Clemants | 2014713 | Wei, Li, Zhang* | Pingtan Fujian | MF063426 | MF063960 | MF065074 | MF064525 | MF073664 | MF073821 |
| *Dysphania ambrosioides* (L.) Mosyakin et Clemants | 2014808 | Wei, Li, Zhang* | Zhangzhou Fujian | MF063427 | MF063961 | MF065075 | MF064526 | MF073665 | MF073822 |
| *Dysphania ambrosioides* (L.) Mosyakin et Clemants | 20141070 | Yao, Zhang | Beihai Guangxi | MF063428 | MF063962 | MF065076 | MF064522 | MF073666 | MF073823 |
| *Dysphania ambrosioides* (L.) Mosyakin et Clemants | 20150003 | Yao, Wei | Zhanjiang Guangdong | MF063429 | MF063963 | MF065077 | MF064527 | MF073667 | MF073824 |
| *Dysphania ambrosioides* (L.) Mosyakin et Clemants | 20150270 | Yao, Wei | Huizhou Guangdong | MF063430 | MF063964 | MF065078 | MF064528 | MF073668 | MF073825 |
| *Dysphania ambrosioides* (L.) Mosyakin et Clemants | 20150502 | Yao, Wei | Wenzhou Zhejiang | - | MF063965 | MF065079 | MF064529 | MF073669 | MF073826 |
| *Dysphania ambrosioides* (L.) Mosyakin et Clemants | 20150655 | Yao, Wei | Wenling Zhejiang | MF063431 | MF063966 | MF065080 | - | MF073670 | MF073827 |
| *Dysphania ambrosioides* (L.) Mosyakin et Clemants | 20150751 | Yao, Wei | Zhoushan Zhejiang | - | MF063967 | MF065081 | MF064530 | MF073671 | MF073828 |
| *Dysphania ambrosioides* (L.) Mosyakin et Clemants |  |  |  | DQ005963 | HE855607 | DQ006049 | HG963733 | HE855681 | HM587682 |
| *Dysphania ambrosioides* (L.) Mosyakin et Clemants |  |  |  | HE577350 | HE855610 | GQ436506 | DQ006134 | HE855682 | - |
| *Dysphania ambrosioides* (L.) Mosyakin et Clemants |  |  |  | HE577351 | HE855611 | HM587599 | HE966568 | HE855683 | - |
| *Dysphania ambrosioides* (L.) Mosyakin et Clemants |  |  |  | HE577352 | HE967380 | HM849889 | HE966567 | HE577493 | - |
| *Dysphania ambrosioides* (L.) Mosyakin et Clemants |  |  |  | HE577353 | HE967381 | - | HE966566 | HE577492 | - |
| *Dysphania ambrosioides* (L.) Mosyakin et Clemants |  |  |  | HE855672 | HE967382 | - | GQ435158 | HE577491 | - |
| *Dysphania ambrosioides* (L.) Mosyakin et Clemants |  |  |  | HE855673 | HM850764 | - | - | HE577488 | - |
| *Dysphania ambrosioides* (L.) Mosyakin et Clemants |  |  |  | HE855674 | KJ772652 | - | - | - | - |
| *Kochia scoparia* (L.) Schrad. | 2014150 | Li, Zhang | Chongming Shanghai | MF063432 | MF063968 |  | MF064531 | MF073673 | MF073830 |
| *Kochia scoparia* (L.) Schrad. | 2014297 | Wei, Li, Zhang | Rongcheng Shandong | MF063433 | MF063969 | MF065082 | MF064532 | MF073674 | MF073831 |
| *Kochia scoparia* (L.) Schrad. | 2014426 | Wei, Li, Zhang | Penglai Shandong | MF063434 | MF063970 | MF065083 | MF064533 | MF073675 | - |
| *Kochia scoparia* (L.) Schrad. | 2014510 | Wei, Li, Zhang | Dongying Shandong | MF063435 | MF063971 | MF065084 | MF064534 | MF073676 | MF073832 |
| *Kochia scoparia* (L.) Schrad. | 2014646 | Wei, Li, Zhang | Ningde Fujian | MF063436 | MF063972 | MF065085 | MF064535 | MF073677 | MF073833 |
| *Kochia scoparia* (L.) Schrad. | 20150515 | Yao, Wei | Wenzhou Zhejiang | MF063437 | MF063973 | MF065086 | MF064536 | MF073678 | MF073834 |
| *Kochia scoparia* (L.) Schrad. | 20150701 | Yao, Wei | Wenling Zhejiang | MF063438 | MF063974 | MF065087 | MF064537 | MF073679 | MF073835 |
| *Kochia scoparia* (L.) Schrad. | 20150794 | Yao, Wei | Zhoushan Zhejiang | MF063439 | MF063975 | MF065088 | MF064538 | MF073680 | MF073836 |
| *Kochia scoparia* (L.) Schrad. | 20151001 | Yao, Li, Wei | Dalian Liaoning | MF063440 | MF063976 | MF065089 | - | MF073681 | MF073837 |
| *Kochia scoparia* (L.) Schrad. | 20151045 | Yao, Li, Wei | Dalian Liaoning | MF063441 | MF063977 | MF065090 | MF064539 | MF073682 | MF073838 |
| *Kochia scoparia* (L.) Schrad. | 20151088 | Yao, Li, Wei | Dalian Liaoning | MF063442 | MF063978 | MF065091 | MF064540 | MF073683 | MF073839 |
| *Kochia scoparia* (L.) Schrad. | 20151117 | Yao, Li, Wei | Dalian Liaoning | MF063443 | MF063979 | MF065092 | MF064541 | MF073684 | MF073840 |
| *Kochia scoparia* (L.) Schrad. | 20151144 | Yao, Li, Wei | Huludao Liaoning | MF063444 | MF063980 | MF065093 | MF064542 | MF073685 | MF073841 |
| *Kochia scoparia* (L.) Schrad. | 20151233 | Yao, Li, Wei | Qinghuangdao Hebei | MF063445 | MF063981 | MF065094 | MF064543 | MF073686 | MF073842 |
| *Kochia scoparia* (L.) Schrad. | 20151318 | Yao, Li, Wei | Lianyungang Jiangsu | MF063446 | MF063982 | MF065095 | MF064544 | MF073687 | MF073843 |
| *Kochia scoparia* (L.) Schrad. | 20151424 | Yao, Li, Wei | Yancheng Jiangsu | MF063447 | MF063983 | MF065096 | MF064545 | MF073688 | MF073844 |
| *Kochia scoparia* (L.) Schrad. | 20160161 | Yao, Wei | Wulumuqi Xinjiang | MF063448 | MF063984 | - | MF064546 | MF073689 | MF073845 |
| *Kochia scoparia* (L.) Schrad. | 20160208 | Yao, Wei | Wulumuqi Xinjiang | MF063449 | MF063985 | MF065097 | MF064547 | MF073690 | MF073846 |
| *Kochia scoparia* (L.) Schrad. | 20160218 | Yao, Wei | Wulumuqi Xinjiang | MF063450 | MF063986 | - | MF064548 | MF073691 | MF073847 |
| *Kochia scoparia* (L.) Schrad. | 20160248 | Yao, Wei | Tulufan Xinjiang | MF063451 | MF063987 | - | MF064549 | MF073692 | MF073848 |
| *Kochia scoparia* (L.) Schrad. | 20160100 | Yao, Li, Wei | Tulufan Xinjiang | MF063452 | MF063988 | MF065098 | MF064550 | MF073693 | MF073849 |
| *Salicornia europaea* L. | 20150435 | Yao, Wei | Nan’ao Guangdong | MF063453 | MF063989 | MF065099 | - | MF073694 | - |
| *Salicornia europaea* L. | 20150841 | Yao, Wei | Zhoushan Zhejiang | MF063454 | MF063990 | MF065100 | MF064552 | MF073695 | MF073851 |
| *Salicornia europaea* L. | 201505076 | Liu | Beihai Guangxi | MF063455 | MF063991 | MF065101 | MF064551 | MF073696 | MF073850 |
| *Salicornia europaea* L. | 20160216 | Yao, Wei | Wulumuqi Xinjiang | MF063456 | MF063992 | MF065102 | MF064553 | MF073697 | MF073852 |
| *Salicornia europaea* L. |  |  |  | AY489247 | - | HM131777 | - | - | - |
| *Salsola komarovii* Iljin | 2014296 | Wei, Li, Zhang | Rongcheng Shandong | MF063457 | MF063993 | MF065103 | MF064554 | MF073698 | MF073853 |
| *Salsola komarovii* Iljin | 2014373 | Wei, Li, Zhang | Penglai Shandong | MF063458 | MF063994 | MF065104 | MF064555 | MF073699 | MF073854 |
| *Salsola komarovii* Iljin | 20151062 | Yao, Li, Wei | Dalian Liaoning | MF063459 | MF063995 | MF065105 | MF064556 | MF073700 | MF073855 |
| *Salsola komarovii* Iljin | 20151070 | Yao, Li, Wei | Dalian Liaoning | MF063460 | MF063996 | MF065106 | MF064557 | MF073701 | MF073856 |
| *Salsola komarovii* Iljin | 20151159 | Yao, Li, Wei | Huludao Liaoning | MF063461 | MF063997 | MF065107 | MF064558 | MF073702 | MF073857 |
| *Salsola komarovii* Iljin | 20151264 | Yao, Li, Wei | Qinghuangdao Hebei | MF063462 | MF063998 | MF065108 | MF064559 | MF073703 | MF073858 |
| *Salsola komarovii* Iljin | 20151369 | Yao, Wei, Yang | Lianyungang Jiangsu | MF063463 | MF063999 | - | MF064560 | MF073704 | MF073859 |
| *Salsola komarovii* Iljin |  |  |  | FJ429355 | - | HM131786 | - | - | - |
| *Salsola komarovii* Iljin |  |  |  | HM131654 | - | - | - | - | - |
| *Salsola tragus* L. | 20151225 | Yao, Li, Wei | Huludao Liaoning | MF063464 | MF064000 | MF065109 | MF064561 | MF073705 | MF073860 |
| *Salsola tragus* L. | 20151307 | Yao, Wei, Yang | Qinghuangdao Hebei | MF063465 | MF064001 | MF065110 | MF064562 | MF073706 | MF073861 |
| *Salsola tragus* L. | 20151209 | Yao, Li, Wei | Huludao Liaoning | MF063466 | - | MF065111 | MF064563 | MF073707 | MF073862 |
| *Salsola tragus* L. |  |  |  | EU373656 | - | HM131795 | - | - | - |
| *Salsola tragus* L. |  |  |  | EU447200 | - | HM131796 | - | - | - |
| *Salsola tragus* L. |  |  |  | HM131652 | - | - | - | - | - |
| *Salsola tragus* L. |  |  |  | KF793935 | - | - | - | - | - |
| *Salsola tragus* L. |  |  |  | HM131667 | - | - | - | - | - |
| *Salsola tragus* L. |  |  |  | HM131668 | - | - | - | - | - |
| *Suaeda australis* (R. Br.) Moq. | 2014513 | Wei, Li, Zhang | Dongying Shandong | MF063467 | MF064002 | MF065112 | MF064564 | MF073708 | MF073864 |
| *Suaeda australis* (R. Br.) Moq. | 2014612 | Wei, Li, Zhang* | Ningde Fujian | MF063468 | MF064003 | MF065113 | MF064565 | MF073709 | MF073865 |
| *Suaeda australis* (R. Br.) Moq. | 2014614 | Wei, Li, Zhang* | Ningde Fujian | MF063469 | MF064004 | MF065114 | MF064566 | MF073710 | MF073866 |
| *Suaeda australis* (R. Br.) Moq. | 2014636 | Wei, Li, Zhang* | Ningde Fujian | MF063470 | MF064005 | MF065115 | MF064567 | MF073711 | MF073867 |
| *Suaeda australis* (R. Br.) Moq. | 2014815 | Wei, Li, Zhang* | Zhangzhou Fujian | MF063471 | MF064006 | MF065116 | MF064568 | MF073712 | MF073868 |
| *Suaeda australis* (R. Br.) Moq. | 20141025 | Yao, Zhang | Beihai Guangxi | MF063472 | MF064007 | MF065117 | MF064569 | MF073713 | MF073869 |
| *Suaeda australis* (R. Br.) Moq. | 20141575 | Yao, Li, Wei | Dongfang Hainan | MF063473 | MF064008 | MF065118 | MF064570 | MF073714 | MF073870 |
| *Suaeda australis* (R. Br.) Moq. | 20150010 | Yao, Wei | Leizhou Guangdong | MF063474 | MF064009 | MF065119 | MF064571 | MF073715 | MF073871 |
| *Suaeda australis* (R. Br.) Moq. | 20150157 | Yao, Wei | Zhanjiang Guangdong | MF063475 | MF064010 | MF065120 | MF064572 | MF073716 | MF073872 |
| *Suaeda australis* (R. Br.) Moq. | 20150261 | Yao, Wei | Huizhou Guangdong | MF063476 | MF064011 | MF065121 | MF064573 | MF073717 | MF073873 |
| *Suaeda australis* (R. Br.) Moq. | 20150434 | Yao, Wei | Nan’ao Guangdong | MF063477 | MF064012 | MF065122 | MF064574 | MF073718 | MF073874 |
| *Suaeda australis* (R. Br.) Moq. | 20150617 | Yao, Wei | Wenling Zhejiang | MF063478 | MF064013 | MF065123 | MF064575 | MF073719 | MF073875 |
| *Suaeda australis* (R. Br.) Moq. | 20150831 | Yao, Wei | Zhoushan Zhejiang | MF063479 | MF064014 | MF065124 | MF064576 | MF073720 | MF073876 |
| *Suaeda australis* (R. Br.) Moq. | 201505073 | Liu | Beihai Guangxi | MF063480 | MF064015 | MF065125 | MF064577 | MF073721 | MF073877 |
| *Suaeda australis* (R. Br.) Moq. |  |  |  | AY181826 | - | - | - | - | AY181826 |
| *Suaeda australis* (R. Br.) Moq. |  |  |  | DQ786334 | - | - | - | - | - |
| *Suaeda australis* (R. Br.) Moq. |  |  |  | DQ786335 | - | - | - | - | - |
| *Suaeda australis* (R. Br.) Moq. |  |  |  | FJ449787 | - | - | - | - | - |
| *Suaeda glauca* (Bunge) Bunge | 20140609 | Yao, Wei | Wenling Zhejiang | MF063481 | MF064016 | MF065126 | MF064581 | MF073726 | MF073881 |
| *Suaeda glauca* (Bunge) Bunge | 2014235 | Yao, Li, Wei | Cixi Zhejiang | MF063482 | MF064017 | MF065127 | MF064578 | MF073723 | MF073878 |
| *Suaeda glauca* (Bunge) Bunge | 2014291 | Wei, Li, Zhang | Rongcheng Shandong | MF063483 | MF064018 | MF065128 | MF064579 | MF073724 | MF073879 |
| *Suaeda glauca* (Bunge) Bunge | 2014374 | Wei, Li, Zhang | Penglai Shandong | MF063484 | - | MF065129 | MF064580 | MF073725 | MF073880 |
| *Suaeda glauca* (Bunge) Bunge | 2014486 | Wei, Li, Zhang | Dongying Shandong | - | MF064019 | - | - | - | - |
| *Suaeda glauca* (Bunge) Bunge | 20150824 | Yao, Wei | Zhoushan Zhejiang | MF063485 | MF064020 | - | MF064582 | MF073727 | MF073882 |
| *Suaeda glauca* (Bunge) Bunge | 20151009 | Yao, Li, Wei | Dalian Liaoning | MF063486 | MF064021 | MF065130 | MF064583 | MF073728 | MF073883 |
| *Suaeda glauca* (Bunge) Bunge | 20151089 | Yao, Li, Wei | Dalian Liaoning | MF063487 | MF064022 | MF065131 | MF064584 | MF073729 | MF073884 |
| *Suaeda glauca* (Bunge) Bunge | 20151147 | Yao, Li, Wei | Huludao Liaoning | MF063488 | MF064023 | MF065132 | MF064585 | MF073730 | MF073885 |
| *Suaeda glauca* (Bunge) Bunge | 20151256 | Yao, Wei, Yang | Qinghuangdao Hebei | MF063489 | MF064024 | MF065133 | MF064586 | MF073731 | MF073886 |
| *Suaeda glauca* (Bunge) Bunge | 20151317 | Yao, Wei, Yang | Lianyungang Jiangsu | MF063490 | MF064025 | MF065134 | MF064587 | MF073732 | MF073887 |
| *Suaeda glauca* (Bunge) Bunge | 20151384 | Yao, Wei, Yang | Yancheng Jiangsu | MF063491 | MF064026 | MF065135 | MF064588 | MF073733 | MF073888 |
| *Suaeda glauca* (Bunge) Bunge |  |  |  | DQ786331 | JF956553 | FJ436013 | JN047267 | FJ436009 | FJ449761 |
| *Suaeda glauca* (Bunge) Bunge |  |  |  | DQ786332 | JF956552 | JF944514 | JN047266 | - | AY181762 |
| *Suaeda glauca* (Bunge) Bunge |  |  |  | DQ786333 | - | JF944515 | - | - | - |
| *Suaeda glauca* (Bunge) Bunge |  |  |  | FJ449825 | - | - | - | - | - |
| *Suaeda glauca* (Bunge) Bunge |  |  |  | JF978816 | - | - | - | - | - |
| *Suaeda glauca* (Bunge) Bunge |  |  |  | JF978817 | - | - | - | - | - |
| *Suaeda salsa* (L.) Pall. | 2014236 | Yao, Li, Wei | Cixi Zhejiang | MF063492 | MF064027 | MF065136 | MF064589 | MF073734 | MF073889 |
| *Suaeda salsa* (L.) Pall. | 2014273 | Wei, Zhang | Fengxian Shanghai | MF063493 | MF064028 | MF065137 | MF064590 | MF073735 | MF073890 |
| *Suaeda salsa* (L.) Pall. | 2014348 | Wei, Li, Zhang | Rongcheng Shandong | MF063494 | MF064029 | MF065138 | MF064591 | MF073736 | MF073891 |
| *Suaeda salsa* (L.) Pall. | 2014488 | Wei, Li, Zhang | Dongying Shandong | MF063495 | MF064030 | MF065139 | MF064592 | MF073737 | MF073892 |
| *Suaeda salsa* (L.) Pall. | 20151120 | Yao, Li, Wei | Dalian Liaoning | MF063496 | MF064031 | MF065140 | MF064593 | MF073738 | MF073893 |
| *Suaeda salsa* (L.) Pall. | 20151216 | Yao, Li, Wei | Huludao Liaoning | - | MF064032 | MF065141 | MF064594 | MF073739 | MF073894 |
| *Suaeda salsa* (L.) Pall. | 20151301 | Yao, Wei, Yang | Qinghuangdao Hebei | MF063497 | MF064033 | MF065142 | MF064595 | MF073740 | MF073895 |
| *Suaeda salsa* (L.) Pall. | 20151338 | Yao, Wei, Yang | Lianyungang Jiangsu | - | MF064034 | MF065143 | MF064596 | MF073741 | MF073896 |
| *Suaeda salsa* (L.) Pall. | 20151401 | Yao, Wei, Yang | Yancheng Jiangsu | MF063498 | MF064035 | MF065144 | MF064597 | MF073742 | MF073897 |
| *Suaeda salsa* (L.) Pall. |  |  |  | DQ899082 | - | - | - | - | AY181763 |
| *Suaeda salsa* (L.) Pall. |  |  |  | DQ899083 | - | - | - | - | FJ449772 |
| *Suaeda salsa* (L.) Pall. |  |  |  | DQ899084 | - | - | - | - | FJ449773 |
| *Suaeda salsa* (L.) Pall. |  |  |  | DQ899085 | - | - | - | - | - |
| *Suaeda salsa* (L.) Pall. |  |  |  | DQ899086 | - | - | - | - | - |
| *Suaeda salsa* (L.) Pall. |  |  |  | DQ899087 | - | - | - | - | - |
| *Suaeda salsa* (L.) Pall. |  |  |  | DQ899088 | - | - | - | - | - |
| *Suaeda salsa* (L.) Pall. |  |  |  | FJ449805 | - | - | - | - | - |
| *Suaeda salsa* (L.) Pall. |  |  |  | KM998386 | - | - | - | - | - |

Note： Li：Hong-Qing Li; Liu：Wen-liang Liu; Wei：Ya-Nan Wei; Yang：Zhu-Ai Yang; Yao：Peng-Cheng Yao; Zhang：Zhen Zhang; Zhang*：Li-Fang Zhang.

Accession numbers begin with “MF” are newly generated in this paper.
