# Supplementary material for: Evaluating sampling strategy for DNA barcoding study of coastal and inland halo-tolerant Poaceae and Chenopodiaceae: A case study for increased sample size
Source: PLoS One. 2017 Sep 21;12(9):e0185311. doi: 10.1371/journal.pone.0185311 (PMC5608404; doi:10.1371/journal.pone.0185311)
Supplement: S3 Table — (DOCX) [file pone.0185311.s005.docx]

**TableS3 Primers information and amplication protocol**

| Sequence |  | (5’-3’) Primers | Amplication Protocol |
| --- | --- | --- | --- |
| ITS | 18sdir  ITS4 | CGTAACAAGGTTTCCGTA  TCCTCCGCTTATTGATATGC  [1] | 94℃4min; 94℃1min; 50℃ 45s; 72℃ 1min 30cycles;  72℃ 5min |
| *mat*K | *mat*K 3F-KIM  *mat*K IR-KIM  *mat*K 390F  *mat*K 1326R | CGTACAGTACTTTTGTGTTTACGAG  ACCCAGTCCATCTGGAAATCTTGGTTC  CBOL(http:// barcoding. si.edu) for Chenopodiaceae  CGATCTATTCATTCAATATTTC  TCTAGCACACGAAAGTCGAAGT  [2] for Poaceae | 94℃1min; 94℃30s; 52℃ 20s; 72℃ 50s 35cycles;  72℃ 5min |
| *rbc*L | *rbc*L a-f  *rbc*L a-r | ATGTCACCACAAACAGAGACTAAAGC  CTTCTGCTACAAATAAGAATCGATCTC  [3] | 95℃4min; 94℃30s; 55℃1min; 70℃ 1min； 94℃ 30s 35cycles;  54℃ 1min |
| *trn*H-*psb*A | *trn*H  *psb*A | CGCGCATGGTGGATTCACAATCC  [4]  GTTATGCATGAACGTAATGCTC  [5] | 95℃4min; 94℃30s; 55℃1min; 72℃ 1min 35cycles;  72℃10min |
| *trn*L-F | *trn*L  *trn*F | GGTTCAAGTCCCTCTATCCC  ATTTGAACTGGTGACACGAG  [6] | 94℃2min; 94℃1min; 52℃1min; 72℃ 2min 30cycles;  72℃15min |
| *atp*B-*rbc*L | *atp*B S2R  *rbc*L1 | AGAAGTAGTAGGATTGATTCTCATA  GAATCCAACACTTGCTTTAGTCTCT  [7] | 94℃2min; 94℃45s; 52℃ 75s; 72℃ 75s 30cycles;  72℃10min |
| *ndh*F | *ndh*F2091R  *ndh*F1311F | GACCCACTCCATTGGTAATTC  ACTGCAGGATTAACTGCGTT  [8] | 95℃4min; 94℃40s; 55℃ 40s; 72℃ 100s 35cycles;  72℃10min |
| *rps*16 | *rps*16F  *rps*16R | AAACGATGTGGTAGAAAGCAAC  ACATCAATTGCAACGATTCGATA  [9] | 95℃3min; 94℃40s; 56℃ 40s; 72℃ 100s 30cycles;  72℃10min |

1. White TJ, Bruns T, Lee S, Taylor J. Amplification and direct sequencing of fungal ribosomal RNA genes for phylogenetics. PCR protocols: a guide to methods and applications. 1990; 18(1):315-322

2. Cuénoud P, Savolainen V, Chatrou LW, Powell M, Grayer RJ, Chase MW. Molecular phylogenetics of Caryophyllales based on nuclear 18S rDNA and plastid *rbc*L, *atp*B, and *mat*K DNA sequences. Am J Bot. 2002; 89(1):132-144 doi: 10.3732/ajb.89.1.132 PMID: 21669721

3. Kress WJ, Erickson DL. A two-locus global DNA barcode for land plants: the coding *rbc*L gene complements the non-coding *trn*H-*psb*A spacer region. PLOS One. 2007; 2(6):e508 doi: 10.1371/journal.pone.0000508 PMID: 17551588

4. Tate JA, Simpson BB. Paraphyly of *Tarasa* (Malvaceae) and diverse origins of the polyploid species. Syst Bot. 2003; 28(4):723-737

5. Sang T, Crawford D, Stuessy T. Chloroplast DNA phylogeny, reticulate evolution, and biogeography of *Paeonia* (Paeoniaceae). Am J Bot. 1997; 84(8):1120 PMID: 21708667

6. Taberlet P, Gielly L, Pautou G, Bouvet J. Universal primers for amplification of three non-coding regions of chloroplast DNA. Plant mol biol. 1991; 17(5):1105-1109 PMID: 1932684

7. Hoot SB, Culham A, Crane PR. The utility of *atp*B gene sequences in resolving phylogenetic relationships: comparison with *rbc*L and 18S ribosomal DNA sequences in the Lardizabalaceae. Ann Mo Bot Gard. 1995. 194-207

8. Romaschenko K, Peterson PM, Soreng RJ, Garcia-Jacas N, Susanna A. Phylogenetics of Stipeae (Poaceae: Pooideae) based on plastid and nuclear DNA sequences. Diversity, Phylogeny, and Evolution in Monocotyledons. Aarhus university press, Denmark. 2010. 511-537

9. Shaw J, Lickey EB, Beck JT, Farmer SB, Liu W, Miller J, et al. The tortoise and the hare II: relative utility of 21 noncoding chloroplast DNA sequences for phylogenetic analysis. Am J Bot. 2005; 92(1):142-166 doi: 10.3732/ajb.92.1.142 PMID: 21652394
